# A Vibration‐Induced‐Emission‐Based Fluorescent Chemosensor for the Selective and Visual Recognition of Glucose

**DOI:** 10.1002/anie.202103545

**Published:** 2021-06-10

**Authors:** Javier Ramos‐Soriano, Sergio J. Benitez‐Benitez, Anthony P. Davis, M. Carmen Galan

**Affiliations:** ^1^ School of Chemistry University of Bristol Cantock's Close Bristol BS8 1TS UK

**Keywords:** DPAC, fluorescent chemosensors, glucose sensing, sugar recognition, vibration-induced emission

## Abstract

The development of chemosensors to detect analytes in biologically relevant solutions is a challenging task. We report the synthesis of a fluorescent receptor that combines vibration‐induced emission (VIE) and dynamic covalent chemistry for the detection of glucose in aqueous media. We show that the bis‐2‐(*N*‐methylaminomethyl)phenylboronic acid‐decorated *N*,*N*′‐diphenyl‐dihydrodibenzo[*a*,*c*]phenazine (DPAC) receptor **1** can detect glucose and discriminate between closely related monosaccharides including those commonly found in blood. Preliminary studies suggest monosaccharides bind to the DPAC‐receptor with a 1:1 stoichiometry to produce pseudomacrocyclic complexes, which in turn leads to distinct optical changes in the fluorescent emission of the receptor for each host. Moreover, the complexation‐induced change in emission can be detected visually and quantified in a ratiometric way. Our results highlight the potential of VIE‐type receptors for the quantitative determination of saccharides in biological samples.

## Graphical abstract

A novel sugar fluorescent chemosensor which combines a vibrational‐induced‐emission (VIE)‐active DPAC core and two *N*‐methylaminomethylphenylboronic acid moieties can detect glucose within seconds and discriminate between closely related monosaccharides including those commonly found in blood.

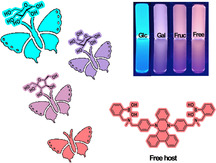

The development of supramolecular chemosensors whereby a synthetic receptor can detect selectively and specifically a given analyte in biologically relevant solutions remains a nontrivial task. In this context, the development of receptors for carbohydrates is particularly challenging due to their structural heterogeneity and polar nature.^[1]^

Carbohydrates are among the most abundant and important molecules in nature. These complex molecules play important roles in nature as fuels (e.g. starch), building materials (e.g. cellulose) and in many physiological and pathological events.^[2]^ Moreover, the development of sensors for the qualitative and quantitative detection of monosaccharides is of great importance for several biomedical purposes.^[3]^ For instance, many researchers have focused their attention on the development of selective glucose receptors for the treatment and prevention of diabetes. These receptors could be exploited in blood glucose monitors^[4]^ and glucose‐responsive insulin.^[5]^ Among the different glucose sensors reported so far, phenylboronic acid‐based molecules, which are known for their ability to form reversible covalent bonds with 1,2‐diols in aqueous media are one of the most studied.^[6]^ The introduction of an aminomethyl moiety in ortho position of the phenylboronic acid enables the recognition event to take place at physiological pH,^[7]^ thus making this system a good candidate for real applications. One of the most remarkable examples is the diphenylboronic anthracene receptor developed by James, Shinkai and co‐workers,^[8]^ which has been recently employed as the recognition unit in a commercially available continuous glucose monitoring device. However, the high similarity in monosaccharide chemical structures, which differ in just the spatial orientation of a certain hydroxyl group (axial vs. equatorial), is one of the main limitations to obtaining sensors with satisfactory selectivities.

Fluorescence has been a widely exploited method for sensing purposes mainly because of its high sensitivity, low cost and the potential for in situ detection. The choice of the fluorophore is influenced by the nature of the receptor, the analyte, and the medium. Recently, Tian and co‐workers disclosed that *N*,*N*′‐disubstituted‐dihydrophenazines show a new fluorescence phenomenon, which relies in a bent‐to‐planar vibration and the reverse in the excited state, called vibration‐induced emission (VIE),^[9]^ that enables the use of this core in ratiometric fluorescence probes. Moreover, Sessler and Stang have developed in parallel, receptors for the discrimination of carboxylic acids based on 9,14‐diphenyl‐9,14‐dihydrodibenzo[*a*,*c*]phe‐nazines (DPAC) that operate in organic solvents.^[10]^ However, as far as we know, the use of VIE‐based receptor for the selective detection of other important analytes in aqueous media has never been described.

Herein we present a new fluorescent monosaccharide receptor that operates in aqueous media, which features a VIE signalling unit tethered via aminomethyl moieties to two phenylboronic acid motifs able to form dynamic covalent bonds with 1,2‐diols. The system can discriminate between very similar compounds e.g., monosaccharides, provoking a striking change in fluorescence that can be monitored by the naked eye. In the case of glucose, the recognition process gives rise to the most drastic change both in colour and increased fluorescence intensity, even in the presence of other monosaccharides. While other systems^[11]^ show comparable selectivities between glucose and other monosaccharides, the current probes’ optical response is not only unique, but easy to monitor by the naked eye. We believe this proof‐of‐concept study demonstrates the potential of this versatile molecules as tools to discriminate between very similar structural analytes in complex biological media.

The synthesis of bi‐functionalized boronic acid **1** and mono‐functionalized **2** were carried out starting from a common intermediate (Scheme 1). Following a modified reported procedure,^[10a]^ Vilsmeier–Haack formylation at the *p*‐position of *N*,*N*′‐diphenyl‐dihydrodibenzo[*a*,*c*]phenazine (DPAC) followed by NaBH_4_ reduction yielded key precursors **3** and **4** bi‐ and monofunctionalized, respectively. Next, the benzylic OH were reacted with 2‐(*N*‐methylaminomethyl)phenylboronic acid pinacol ester in presence of [Ru(*p*‐cymene)Cl_2_]Cl_2_, DPEPhos and Na_2_CO_3_ to give the corresponding tertiary amines **1** and **5** from **3** and **6** from **4** by borrowing hydrogen catalysis.^[12]^ Boronic derivatives **1** and **2** were thus subsequently obtained in 90–94 % yields, after the removal of pinacol ester groups. The structures of both **1** and **2** and their corresponding intermediates were confirmed by ^1^H NMR and ^13^C NMR spectroscopy, as well as high‐resolution mass spectrometry (see ESI).

**Scheme 1:**
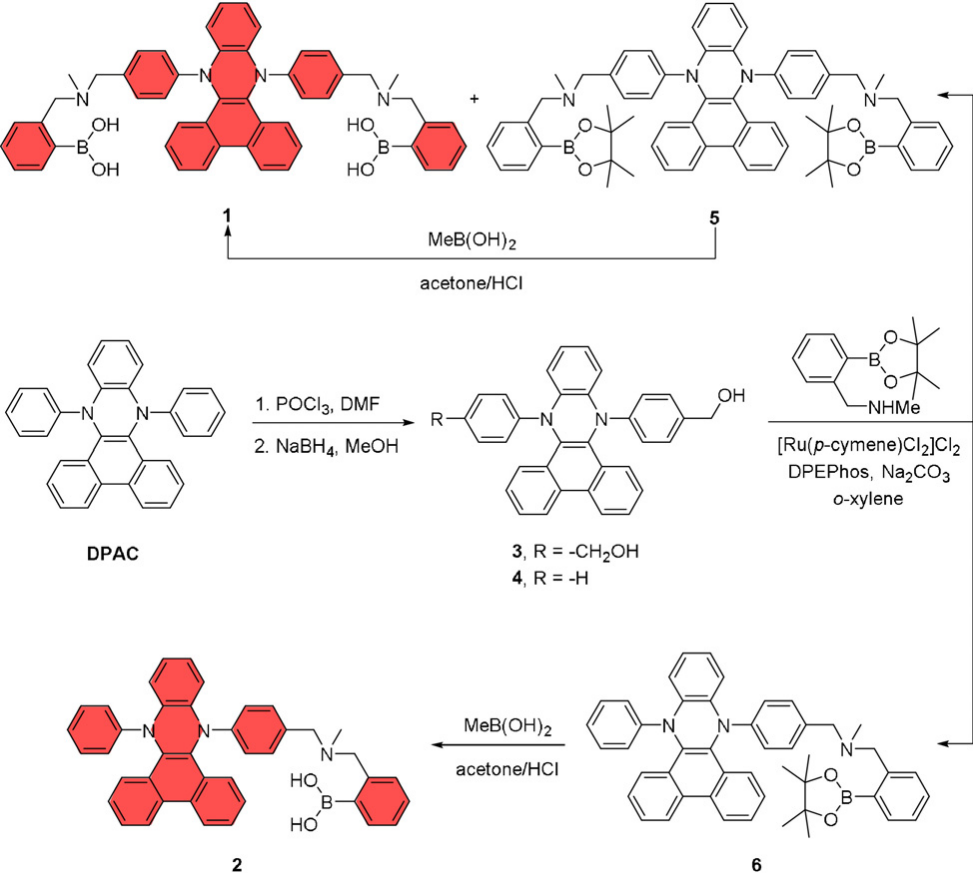
Synthesis of the receptor **1** and the control compound **4**.

Initial binding studies of **1** with a range of common monosaccharides such as d‐glucose, d‐fructose and d‐galactose, which are the main monosaccharides present in blood,[^8a^, ^13^] were carried out by fluorescence spectroscopy titrations in 80 % methanol/10 mM phosphate buffer (pH 7.4) solutions to allow the solubilization of both the sugar and **1**. The emission spectrum of **1** when excited at 350 nm, in the absence of ligand, showed an intense broad band at 592 nm, along with small emission band around 432 nm, consistent with previous studies of DPAC derivatives.^[10a]^ Excitingly, the addition of d‐glucose gave rise to the appearance of a new very intense band at 452 nm with concomitant decrease of the band at 592 nm (Figure 1 a and Figure S1). An isosbestic point was observed at 579 nm, suggesting the presence of two different species, complex and free receptor, in equilibrium. The entire process can be readily visualized by the naked eye as the emission profile of the solution changes from an orange‐reddish colour (free receptor) to a bright turquoise tone (saturated system) (Figure 1 d and S1). Next, addition of d‐galactose to **1** decreased the intensity of the lower‐energy band, albeit the decrease was less dramatic when compared to the d‐glucose substrate, while a new higher‐energy emission band at 450 nm appeared. The maximum of this band was slightly blue shifted in comparison to the new band observed with d‐glucose and in general exhibited a lower intensity at the same concentrations, giving rise to a purplish colour at saturation (Figure 1 b and Figure S2). In the case of d‐fructose, a new band centred at 444 nm appeared (Figure 1 c and Figure S3), although with a lower emission than in the two cases aforementioned, and saturation of the system was reached at a lower sugar/receptor concentration ratio, suggesting the higher affinity of **1** for d‐fructose of the triad studied. Overall, the addition of d‐fructose led to an apparent fluorescence quenching with no observable change in the colour of the free receptor. The reasons for these effects are currently under investigation.

**Figure 1:**
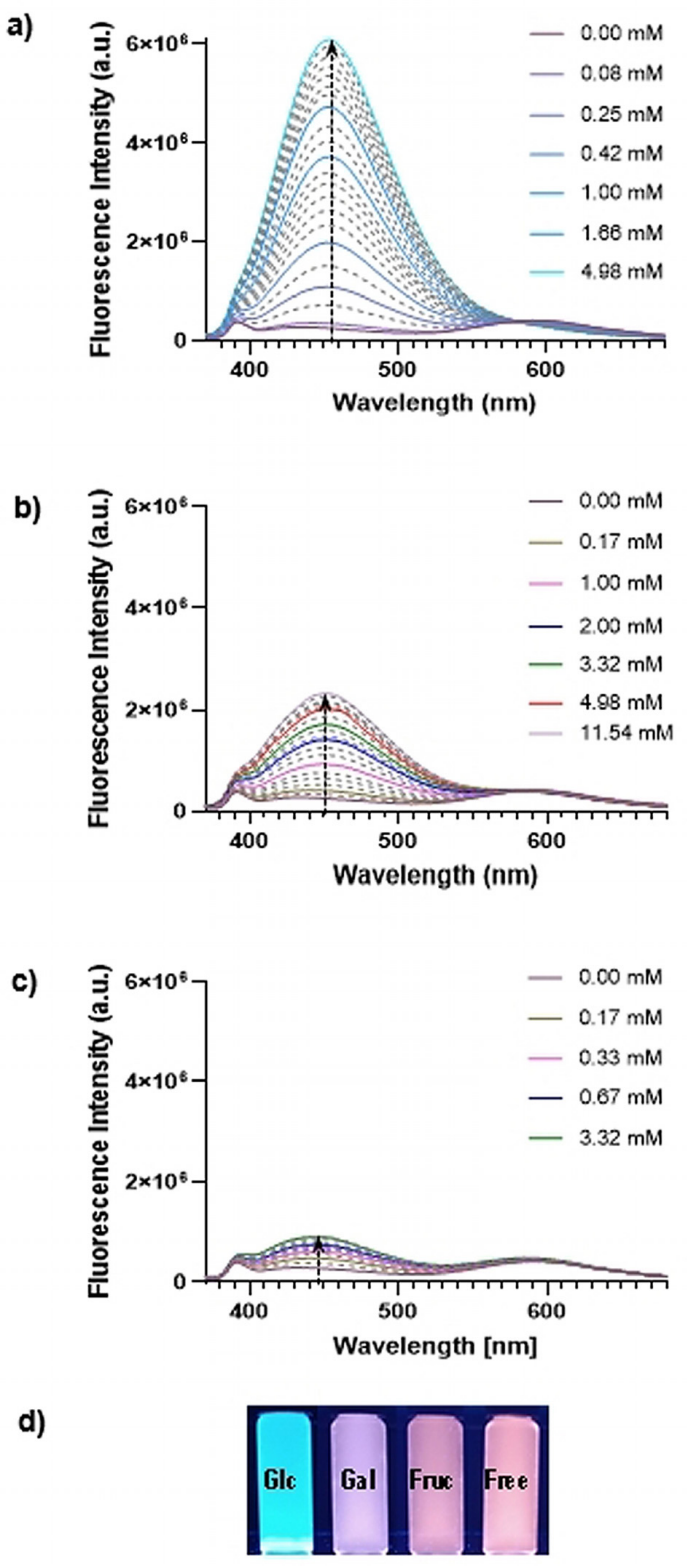
Fluorescence emission spectral changes seen upon the addition of increasing quantities of a) d‐glucose, b) d‐galactose and c) d‐fructose to a solution of **1** (*λ*_ex_=350 nm). d) Fluorescence images of **1** (free) and **1**⊃sugars recorded upon addition with 365 nm UV light, [**1**]=1 μM, solvent: MeOH/10 mM phosphate buffer (80:20).

These results demonstrate that the fluorescence response of **1** upon ligand binding is characteristic for each sugar, allowing the discrimination between d‐glucose, d‐galactose and d‐fructose even by the naked eye. Moreover, the recognition event can be monitored in a ratiometric way, which endows our system the potential to be used in the development of tools for the quantitative determination of saccharides in aqueous media.

In order to study the selectivity of our receptor for d‐glucose, we performed qualitative fluorescence competition titrations. Increasing amounts of d‐glucose were added to already saturated systems containing **1** and either d‐galactose or d‐fructose, respectively. In both cases, the addition of d‐glucose provoked a bathochromic shift to 452 nm, which is the characteristic band for the **1**⊃d‐glucose complex and a significant increase in intensity. For the complex **1**⊃d‐galactose, this band shifted from 450 nm to 452 nm when increasing concentrations of d‐glucose were added (0–65 mM) (Figure 2 a), whereas the shift for the complex **1**⊃d‐fructose was more dramatic from 444 to 452 nm upon addition of 0–10 mM of d‐glucose (Figure 2 b). These changes were also visible in the fluorescence emission color which shifted from either purple (**1**⊃d‐galactose) and orange‐red (**1**⊃d‐fructose) to a bright turquoise shade (**1**⊃d‐glucose), indicating that the fluorescence emission for the glucose complex overwhelms that from the fructose or galactose complexes in solution.

**Figure 2:**
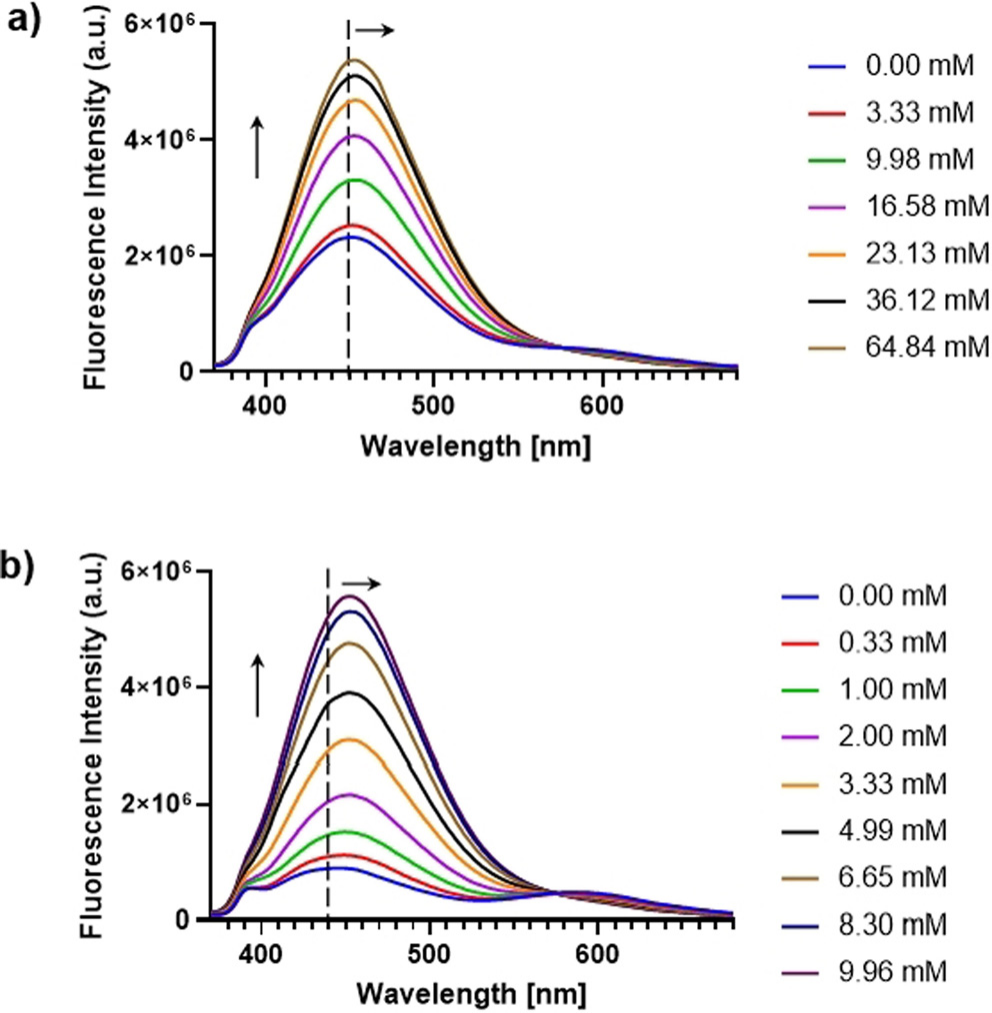
Fluorescence competition titrations of a) **1**⊃d‐galactose ([d‐galactose]=11.54 mM) and b) **1**⊃d‐fructose ([d‐fructose]=3.32 mM) with d‐glucose. *λ*_ex_=350 nm, [**1**]=1 μM, solvent=MeOH/10 mM phosphate buffer (80:20).

The concentration of free glucose in biological samples is typically higher than that of other monosaccharides[^8a^, ^13^] and thus the receptor developed herein offers great potential as a glucose sensor for real‐world applications. To demonstrate this practically, we examined the response of **1** in the presence of an equimolar mixture of d‐glucose, d‐galactose and d‐fructose in real time (see video in SEI). For this purpose, the sugar mixture was added dropwise to a solution of **1** under UV light irradiation (365 nm). Within seconds, a change of colour from orange‐reddish to bright turquoise was observed, which is in accordance with the results of the competitive titrations.

To evaluate the versatility and selectivity of the system, other less common monosaccharides were also investigated as ligands. The addition of d‐mannose, l‐arabinose, l‐fucose, d‐xylose or l‐rhamnose to **1** also induced the appearance of new emission bands at 440, 447, 453, 444 and 433 nm, respectively (Figure 3 and Figures S4–S10). The fluorescence emission and intensity were distinct for each saccharide with different colours obtained for the saturated systems, and more importantly none of them exhibit the same emission profile as the **1**⊃d‐glucose complex (Figure 3). Competition titrations between the different **1**⊃sugar complexes and d‐glucose were also conducted, with similar results as observed for the galactose and fructose complexes. (Figures S4–S8). Interestingly, when the binding of **1** and *N*‐acetyl‐β‐d‐glucosamine or methyl‐β‐d‐glucopyranoside were investigated, no significant changes were observed in the emission spectra for **1**. These findings clearly show the need for the carbohydrate molecule to bear free hydroxyl groups at C‐1 and C‐2 positions for effective interaction with **1** and that this receptor is ideal for detecting free glucose in the presence of glucosides.

**Figure 3:**
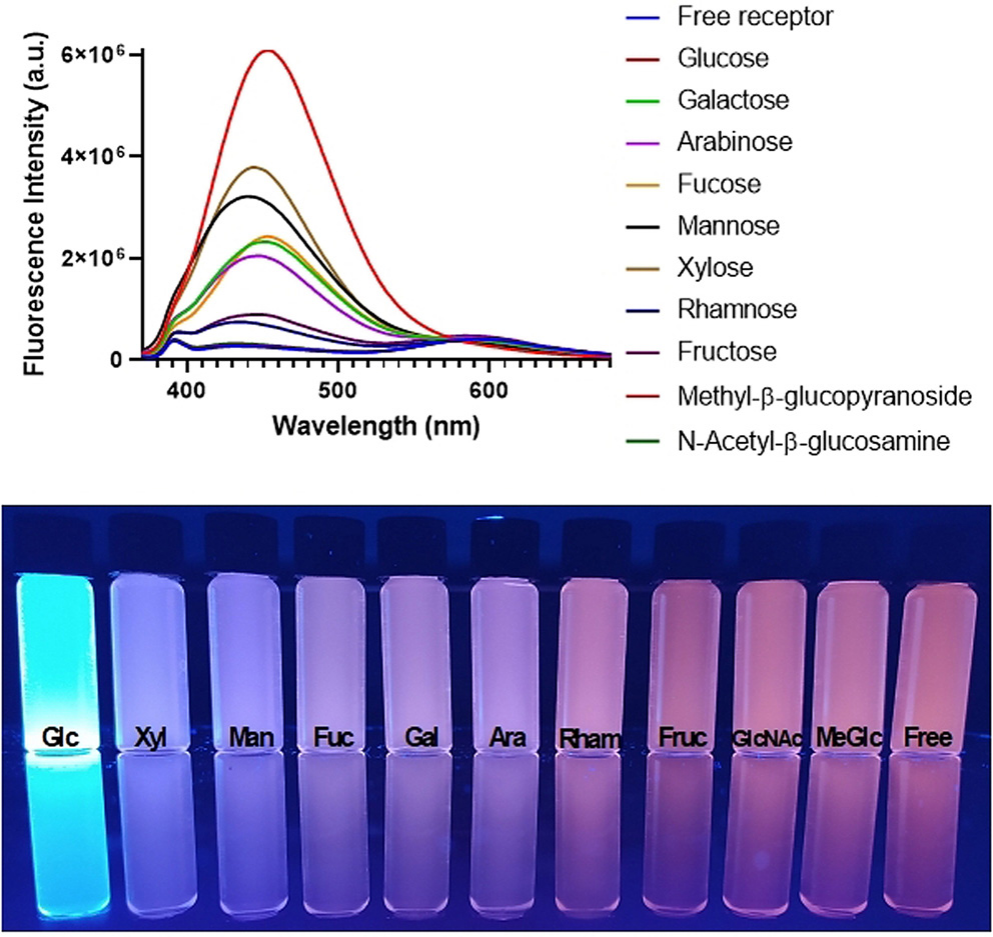
Fluorescence emission spectra of **1** in the presence (as well as absence) of various monosaccharides at the end of titrations associated with fluorescence image under irradiation with 365 nm UV light. *λ*_ex_=350 nm, [**1**]=1 μM, solvent=MeOH/10 mM phosphate buffer (80:20).

Based on previously reported binding data for DPAC‐based receptors with dicarboxylate dianions,^[10]^ we hypothesised that the changes observed in the emission spectra of diboronic receptor **1** upon the addition of monosaccharides is likely a consequence of the formation of a 1:1 complex. Vibration‐induced planarization of the excited state of the fluorophore would be impeded due to the constraint imposed by the formation of dynamic covalent bonds between the two phenyl boronic moieties and the monosaccharide, giving rise to the observed hypsochromic shift in the emission. This is further supported by experiments carried out in either pure MeOH or MeOH/10 mM phosphate buffered‐D_2_O (80:20) mixtures, whereby a similar emission profile for the complex is observed upon glucose binding to **1** as in experiments carried out in the presence of H_2_O (Figures S38,S39 in ESI), discarding a “loose bolt effect” internal conversion mechanism exhibited by other boronic acid‐based sensors.^[11d]^

To gain a better understanding into the proposed receptor/ligand interaction, control experiments were conducted with monotopic receptor **2**. As expected, negligible changes in the emission spectrum of **2** were observed upon addition of any of the sugars (Figures S11–S20). These results highlight the need for the receptor to bear two recognition centres to exploit the remarkable optical properties of the DPAC‐based fluorophore in the recognition of monosaccharides. In addition, the binding constants of receptor **1** towards the different monosaccharides studied were obtained by fluorescence titration and are listed in Table 1. We used the supramolecular.org Web applet to fit the original data. In all the cases, good fitting was obtained for a 1:1 stoichiometric binding model (see Figures S21–S28 of the SEI for details of the fitting). As expected for a diphenylboronic acid‐based sugar receptor, compound **1** showed a higher affinity towards d‐fructose (*K*_a_=1927 m ^−1^) and d‐glucose (*K*_a_=816 m ^−1^) over the other monosaccharides. The stoichiometry for the complex **1**⊃d‐glucose was further supported by mass spectrometry (Figure S29), in which the (M+H)^+^ ion was the higher peak (*m*/*z* 897.4).^[14]^

**Table 1:** Binding constants and limits of detection (LODs) for receptor **1** and monosaccharides guests in MeOH/10 mM phosphate buffer (80:20) at 298 K.

Monosaccharides	Binding Constants [m ^−1^]	LOD [μM]
d‐Glucose	K_1:1_=816	9.4
d‐Galactose	K_1:1_=400	63.1
d‐Fructose	K_1:1_=1927	40.3
d‐Xylose	K_1:1_=593	17.2
d‐Mannose	K_1:1_=395	45.6
l‐Arabinose	K_1:1_=339	74.2
l‐Fucose	K_1:1_=137	128.1
l‐Rhamnose	K_1:1_=53	2038.8
Methyl‐β‐d‐glucose	NB^[a]^	–
*N*‐Acetyl‐β‐d‐glucosamine	NB^[a]^	–

[a] Not binding. Errors below 10 %.

The limit of detection (LODs) for the different monosaccharides studied was also calculated (Table 1 and Figures S30–37) from the fluorescence data, showing the lowest value for d‐glucose (9.4 μM). This is not unexpected as the **1**⊃d‐glucose exhibit the biggest increase in emission intensity and fluorescent emission shift of all the complexes evaluated.

In summary, we have successfully developed a sugar fluorescent chemosensor **1** which combines a VIE‐active DPAC core and two *N*‐methylaminomethylphenylboronic acid moieties. The system is able to discriminate between similar monosaccharides for example, d‐glucose over d‐galactose and d‐fructose which are the three main monosaccharides found in blood, as well as other related monosaccharides, by displaying a change in fluorescence upon ligand binding that can be monitored by the naked eye. A combination of fluorescent spectral curve fittings, mass spectrometry and control experiments provided support for the conclusion that monosaccharide guests considered in the present study bind to **1** with a 1:1 stoichiometry. Moreover, the recognition event can be monitored in a ratiometric manner, allowing the identification of d‐glucose in the presence of other monosaccharides in just a few seconds in real time (see video in ESI). This proof of concept study is the first example, to the best of our knowledge, of a DPAC‐based receptor that discriminates sugars in aqueous media and opens the door to the development of improved tools for the quantitative determination of saccharides in biological samples. Further studies are under active exploration in our laboratory and will be reported in due course.

## Conflict of interest

The authors declare no conflict of interest.

## Supporting information

As a service to our authors and readers, this journal provides supporting information supplied by the authors. Such materials are peer reviewed and may be re‐organized for online delivery, but are not copy‐edited or typeset. Technical support issues arising from supporting information (other than missing files) should be addressed to the authors.

Supplementary

Supplementary
