# Supplementary material for: A Vibration‐Induced‐Emission‐Based Fluorescent Chemosensor for the Selective and Visual Recognition of Glucose
Source: Angew Chem Int Ed Engl. 2021 Jun 10;60(31):16880–4. doi: 10.1002/anie.202103545 (PMC8362141; doi:10.1002/anie.202103545)
Supplement: Supplementary file 1 — Supplementary [file ANIE-60-16880-s001.pdf]

Angewandte Chemie
Eine Zeitschrift der Gesellschaft Deutscher Chemiker

Supporting Information

A Vibration-Induced-Emission-Based Fluorescent Chemosensor for the Selective and Visual Recognition of Glucose

Javier Ramos-Soriano, Sergio J. Benitez-Benitez, Anthony P. Davis, and M. Carmen Galan**

anie_202103545_sm_miscellaneous_information.pdf
anie_202103545_sm_Video.mp4

Supporting Information

1. General *S2*
2. Synthesis and characterization *S3*
3. Fluorescent response of **1** to D-glucose, D-fructose and D-galactose *S11*
4. Fluorescent response of **1** to other monosaccharides *S13*
5. Fluorescent response of **4** to monosaccharides *S19*
6. Titrations fitting and binding isotherms *S24*
7. MS spectrum of complex **1**⊃D-glucose *S28*
8. Determination of the limit of detection (LOD) *S29*
9. Fluorescence response of **1** to D-glucose in pure MeOH and MeOH/10mM phosphate buffered-D_2O (80:20) *S33*
10. Reference *S34*

1. General

Synthesis and characterization. Reagents and solvents were purchased as reagent grade and used without further purification. Compounds **DPAC, 3** and **4** were prepared according to previously reported procedures.[1] For column chromatography, silica gel 60 (230-400 mesh, 0.040-0.063 mm) was purchased from E. Merck. Thin Layer Chromatography (TLC) was performed on aluminium sheets coated with silica gel 60 F254 purchased from E. Merck, visualization by UV light. NMR spectra were recorded on a Bruker AC 400 with solvent peaks as reference. ^{1}H and ^{13}C NMR spectra were obtained for solutions in CD_3OD. All the assignments were confirmed by one- and two-dimensional NMR experiments (DEPT, COSY, HSQC and HMBC). Mass spectra were obtained by the University of Bristol mass spectrometry service by electrospray ionisation (ESI).

Binding studies. All fluorescence titration experiments were carried out at 298 K on a Horiba Fluoromax spectrofluorometer. A solution of the receptor (3 mL) at a known concentration (1 μM) in methanol/10 mM phosphate buffer (80:20), was placed in a quartz cuvette (3 mL, 10 mm path length). A solution of the desired saccharide in phosphate buffer solution (pH 7.4, 10 mM) was then added to the solution containing the receptor and the fluorescence spectrum was recorded after each addition. Guest additions to the receptor were performed using a procedure which kept the concentration of receptor constant throughout the titration. The excitation wavelength was fixed at 350 nm for each titration, and the emission spectrum recorded between 400 – 700 nm. The binding constants alongside their standard deviation were calculated by a non-linear least squares fitting using the online tool Bindfit (http://app.supramolecular.org/bindfit/). All fluorescence titration experiments were carried out three times.

2. Synthesis and Characterization.

Synthesis of compound 1.

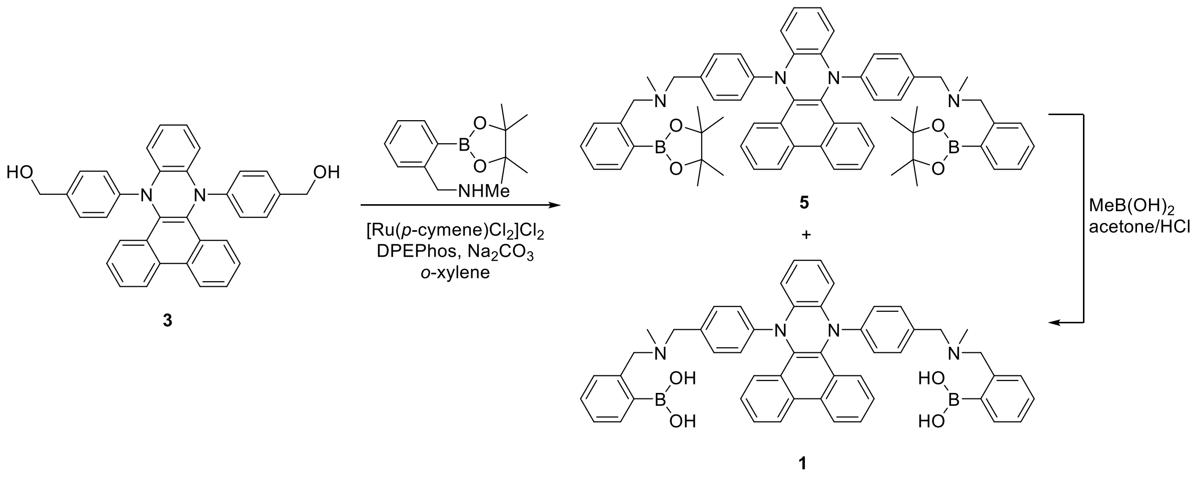

To a nitrogen-purged microwave tube containing **3** (200 mg, 0.40 mmol), 2-(*N*-methylaminomethyl)phenylboronic acid picanol ester (204 mg, 0.80 mmol), [Ru(*p*-cymene)Cl_2]$_2$ (12.4 mg, 0.02 mmol), DPEPhos (44 mg, 0.08 mmol), Na_2CO_3 (8.6 mg, 0.08 mmol) and dry *o*-xylene (2 mL). The reaction mixture was allowed to stir under a nitrogen atmosphere at room temperature and then increased the temperature to 155 °C to reflux for 24h. After that, the resulting crude compounds were evaporated *in vacuo*. The residue was purified by flash silica chromatography ($CHCl_3$/MeOH/TFA, 9:1:0.05 → 5:1:0.05), afforded compounds **5** (136 mg, 35%) and **1** (109 mg, 34%) as yellowish amorphous solids. To a small round-bottom flask, pinacol ester derivative **5** (100 mg, 0.10 mmol) and methylboronic acid (64 mg, 1.05 mmol) were added and dissolved in acetone/0.2 m HCl (4 mL, 1:1, v/v). The reaction mixture was stirred at room temperature overnight. Upon completion of the n reaction as shown by TLC, the reaction mixture was concentrated to dryness, redissolved in $CHCl_3$ and dried *in vacuo*. The residue was purified by flash silica chromatography ($CHCl_3$/MeOH/TFA, 5:1:0.05), afforded compound **1** (83 mg, 90%) as a yellowish amorphous solid.

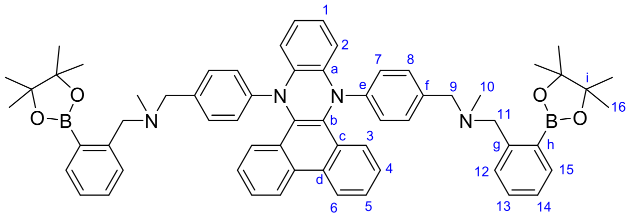

5: ^{1}H NMR (400 MHz, MeOD-*d*$_4$) δ 8.84 (d, *J* = 8.4 Hz, 2H, H-6), 8.01 (dd, *J* = 8.2, 1.3 Hz, 2H, H-3), 7.93 (dd, *J* = 7.4, 1.6 Hz, 2H, H-15), 7.89 – 7.85 (m, 2H, H-2), 7.69 (t, *J* = 7.6 Hz, 2H, H-5), 7.57 – 7.51 (m, 4H, H-4 and H-13), 7.50 – 7.44 (m, 4H, H-1 and H-14), 7.39 (dd, *J* = 7.5, 1.3 Hz, 2H, H-12), 7.15 (d, *J* = 8.8 Hz, 4H, H-7),

7.03 (d, J = 8.8 Hz, 4H, H-8), 4.63 (m, 2H, H-11a), 4.36 – 4.19 (m, 4H, H-11b and H-9a), 4.12 (m, 2H, H-9b), 2.52 (s, 6H, H-10), 1.32 (s, 24H, H-16); ^{13}C NMR (101 MHz, MeOD-d_4) δ 150.0 (C-e), 145.4 (C-a), 139.0 (C-b), 138.4 (C-15), 136.7 (C-g), 133.2 (C-7), 133.1 (C-13), 133.0 (C-12), 131.3 (C-d and C-h), 130.5 (C-14), 129.9 (C-c), 128.9 (C-2), 128.3 (C-4 + C-5), 127.4 (C-1), 125.1 (C-3), 124.6 (C-6), 122.0 (C-f), 117.4 (C-8), 86.0 (C-i), 60.9 (C-9), 60.0 (C-11), 38.9 (C-10), 25.4 - 25.0 (C-16); ESI-HRMS for $C_{62}H_{67}B_2N_4O_4$ $[M + H]^+$ calcd: 953.5362, found: 953.5368.

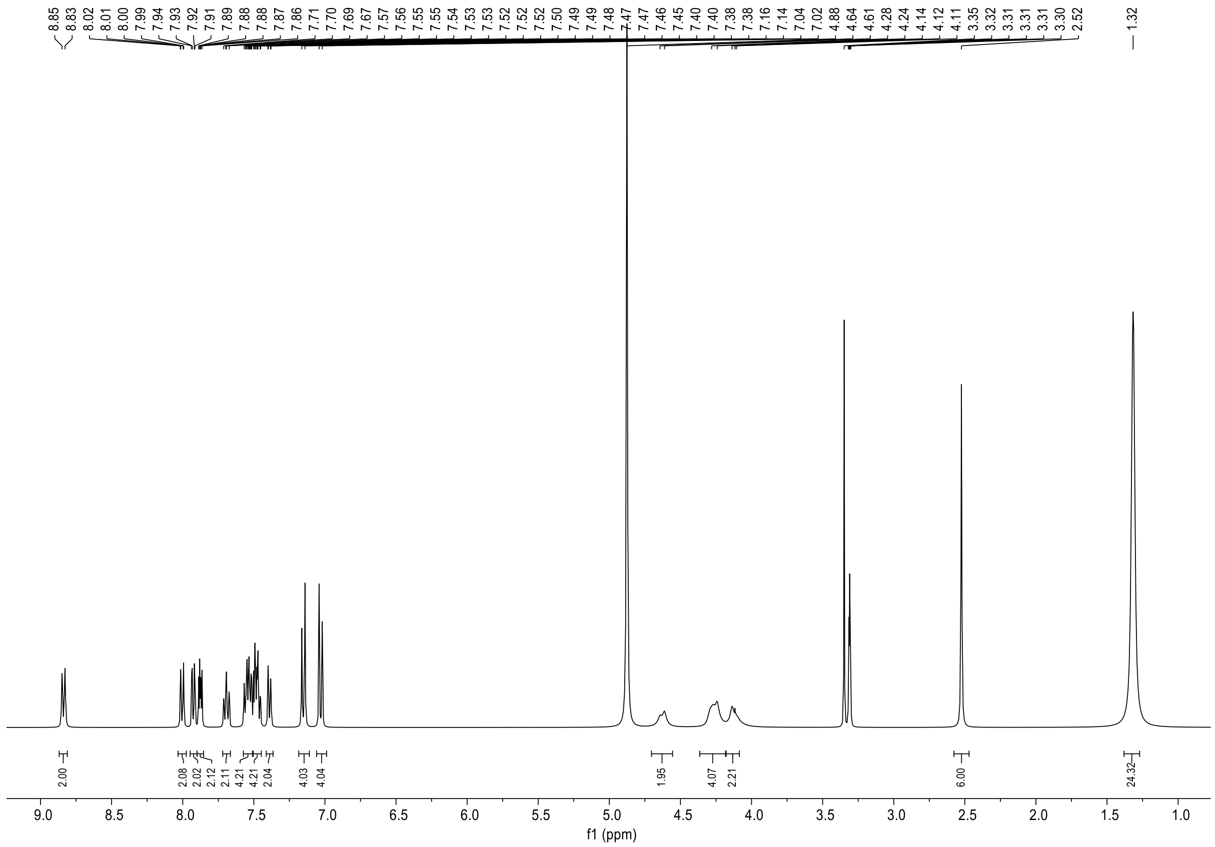

^{1}H NMR spectrum of compound **5** (CD_3OD, 400 MHz).

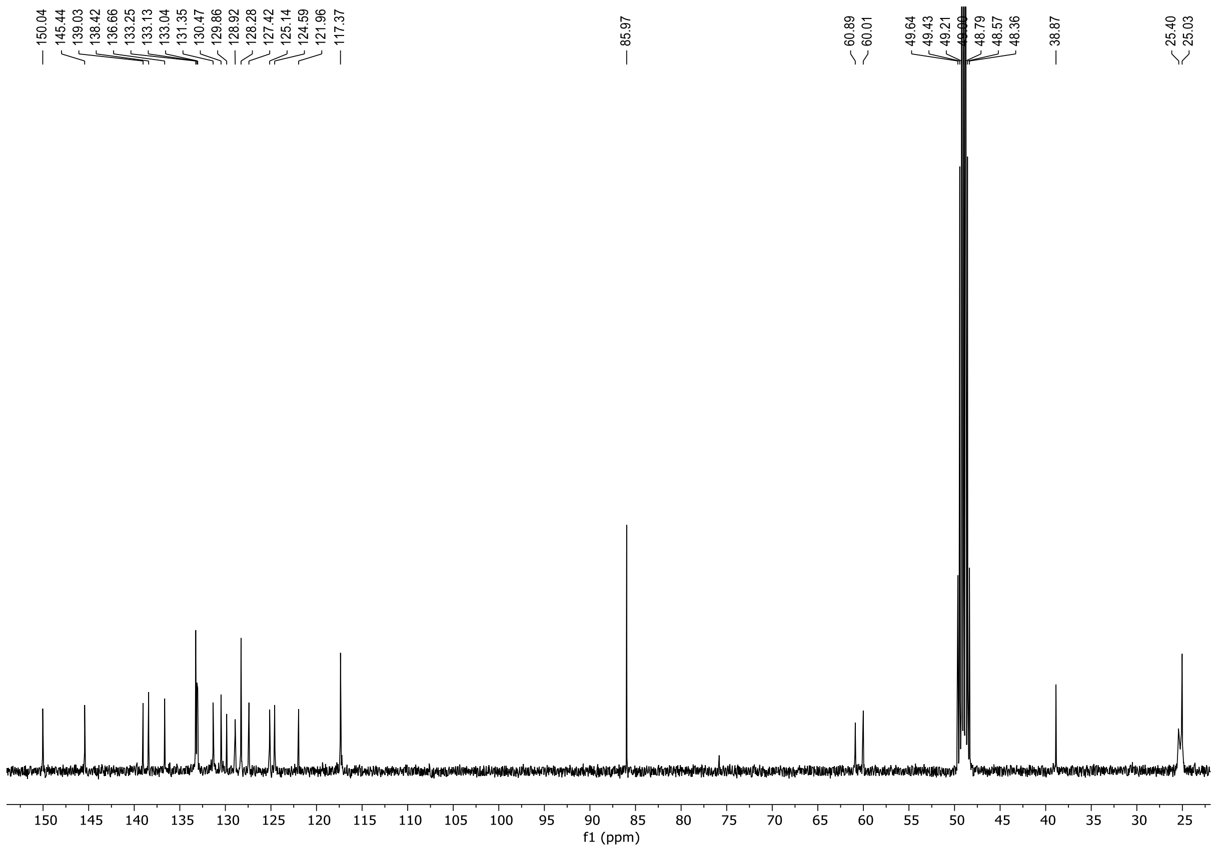

^{13}C NMR spectrum of compound **5** (CD_3OD, 100 MHz).

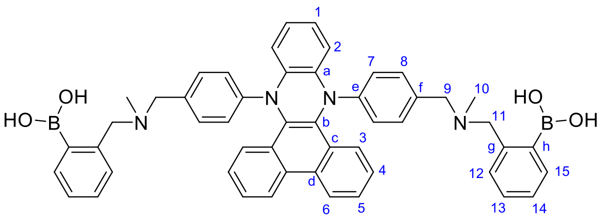

1: ^{1}H NMR (400 MHz, MeOD-d_4) δ 8.87 (d, J = 8.3 Hz, 2H, H-6), 8.02 (dd, J = 8.2, 1.3 Hz, 2H, H-3), 7.89 (dd, J = 5.9, 3.4 Hz, 2H, H-2), 7.80 – 7.67 (m, 4H, H-5 and H-15), 7.57 (ddd, J = 8.2, 7.0, 1.0 Hz, 2H, H-4), 7.49 (dd, J = 5.9, 3.4 Hz, 2H, H-1), 7.44 (dd, J = 5.6, 3.3 Hz, 4H, H-13 and H-14), 7.33 (dd, J = 5.5, 3.4 Hz, 2H, H-12), 7.15 (d, J = 8.9 Hz, 4H, H-7), 7.02 (d, J = 8.9 Hz, 4H, H-8), 4.44 – 3.93 (m, 8H, H-9 and H-11), 2.46 (s, 6H, H-10); ^{13}C NMR (101 MHz, MeOD-d_4) δ 149.9 (C-e), 145.5 (C-a), 139.1 (C-b), 136.5 (C-h), 135.5 (C-g), 133.2 (C-12), 133.0 (C-7), 131.4 (C-13), 131.3 (C-d), 130.2 (C-14), 129.9 (C-c), 129.0 (C-2), 128.2 (C-4, C-5 and C-15), 127.4 (C-1), 125.1 (C-3), 124.5 (C-6), 122.3 (C-f), 117.1 (C-8), 61.1, 61.0 (C-9 and C-11), 39.1 (C-10); ESI-HRMS for $C_{50}H_{45}B_2N_4O_3$ $[M - H_2O + H]^+$ calcd: 771.3678, found: 771.3687.

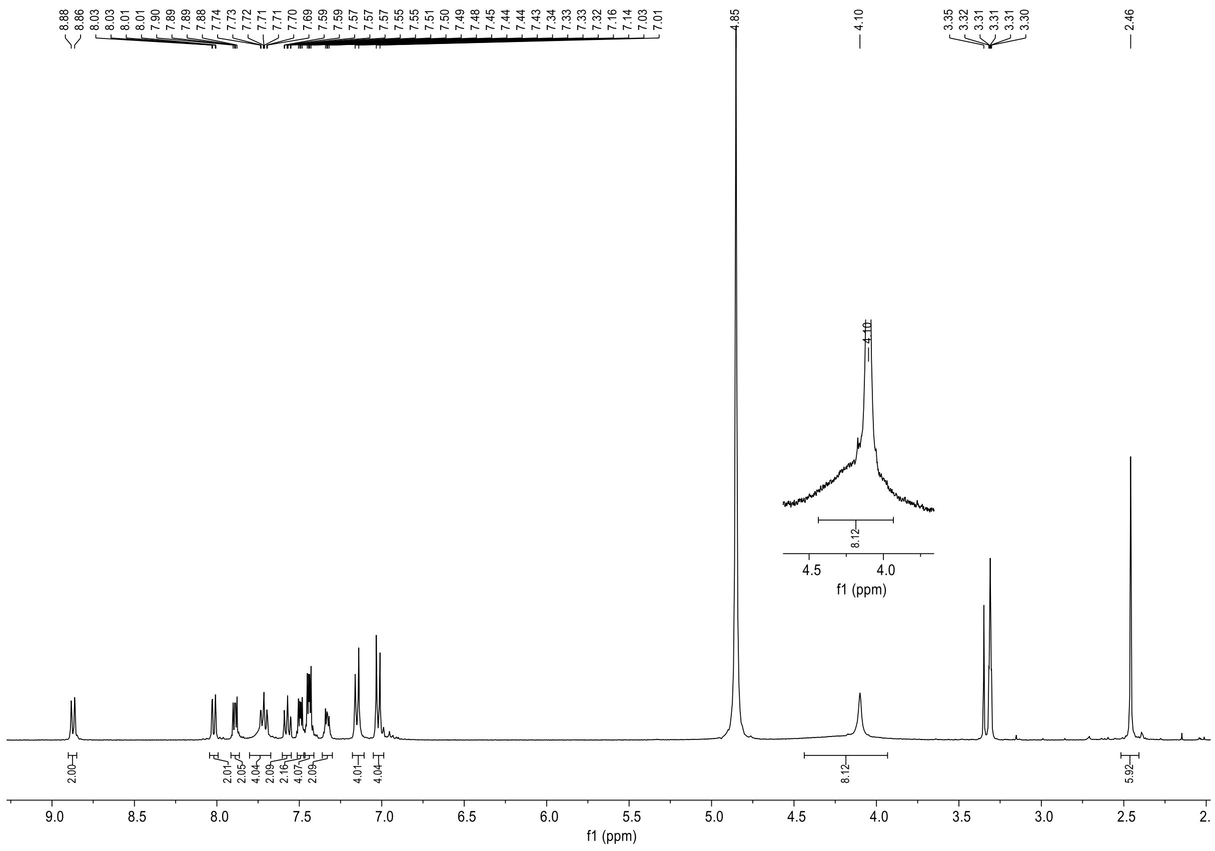

^{1}H NMR spectrum of compound **1** (CD_3OD, 400 MHz).

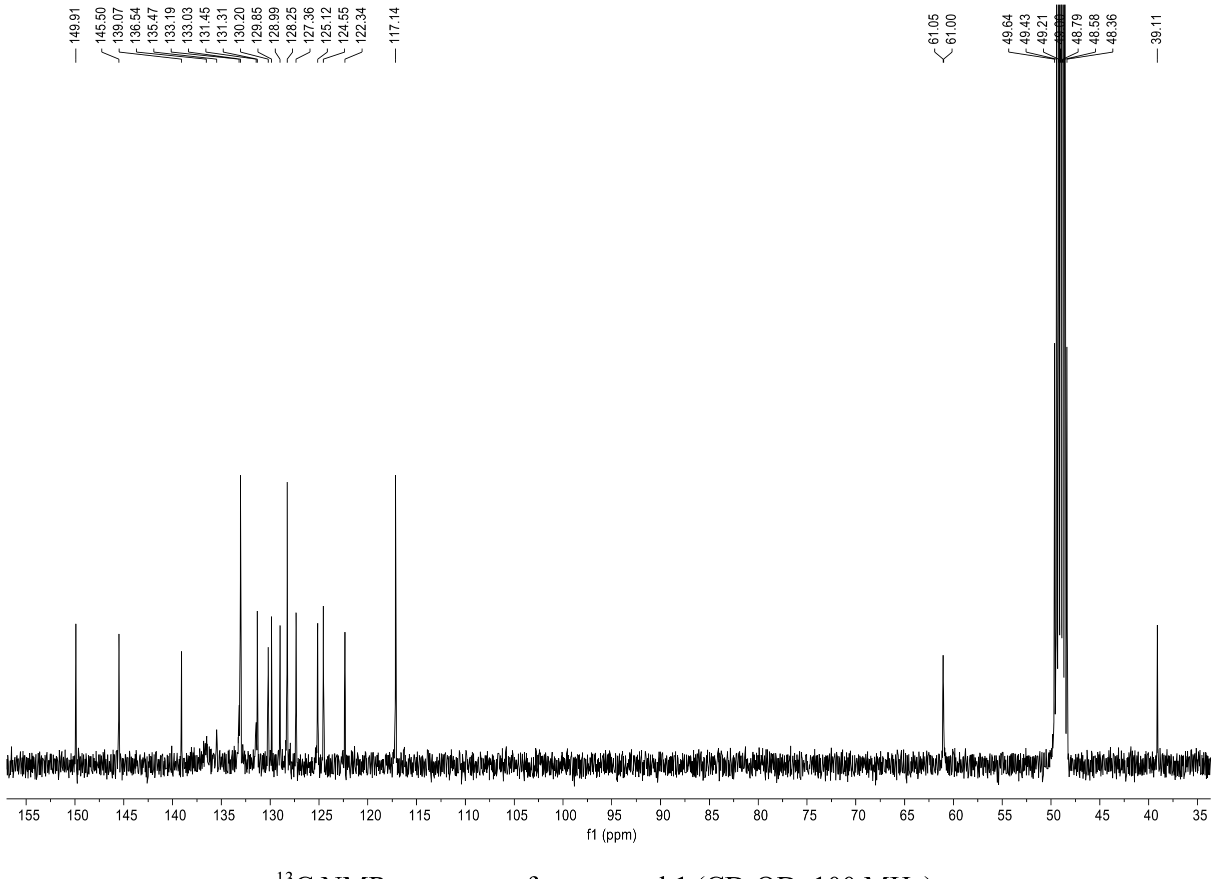

^{13}C NMR spectrum of compound **1** (CD_3OD, 100 MHz).

Synthesis of compound 6.

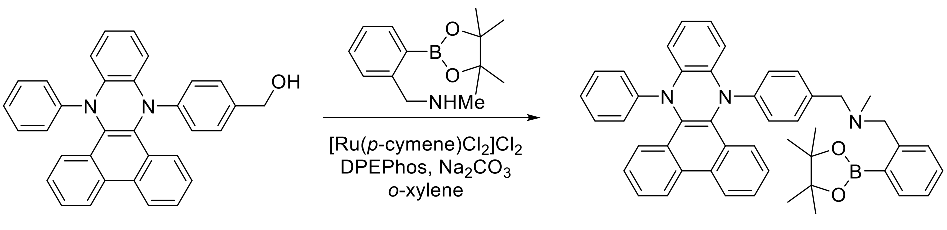

To a nitrogen-purged microwave tube containing **4** (150 mg, 0.32 mmol), 2-(*N*-methylaminomethyl)phenylboronic acid picanol ester (81.5 mg, 0.32 mmol), [Ru(*p*-cymene)Cl_2]$_2$ (9.9 mg, 0.02 mmol), DPEPhos (35 mg, 0.06 mmol), Na_2CO_3 (6.8 mg, 0.06 mmol) and dry *o*-xylene (2 mL). The reaction mixture was allowed to stir under a nitrogen atmosphere at room temperature and then increased the temperature to 155 °C to reflux for 24h. After that, the resulting crude mixture was evaporated *in vacuo*. The residue was purified by flash silica chromatography ($CHCl_3$/MeOH/TFA, 9:1:0.05 → 5:1:0.05) to afford compound **6** (146 mg, 65%) as a yellowish amorphous solid.

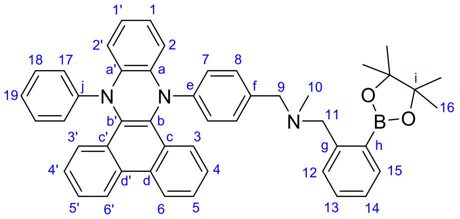

^{1}H NMR (400 MHz, $CDCl_3$) δ 8.75 (d, J = 8.3 Hz, 2H, H-6 and H-6'), 8.16 (ddd, J = 10.6, 8.2, 1.4 Hz, 2H, H-3 and H-3'), 7.80 – 7.72 (m, 2H, H-2 and H-2'), 7.66 (dddd, J = 8.3, 7.0, 2.5, 1.4 Hz, 2H, H-5 and H-5'), 7.61 – 7.53 (m, 3H, H-4, H-4' and H-15), 7.39 – 7.34 (m, 2H, H-1 and H-1'), 7.24 – 7.18 (m, 2H, H-13 and H-14), 7.04 (br s, 1H, H-12), 7.01 – 6.92 (m, 4H, H-17 and H-18), 6.88 (br s, 4H, H-7 and H-8), 6.74 (t, J = 6.9, 1H, H-19), 3.66 – 3.38 (m, 4H, H-9 and H-11), 2.14 (s, 3H, H-10), 1.29 (s, 12H, H-16); ^{13}C NMR (101 MHz, $CDCl_3$) δ 148.0 (C-e), 147.8 (C-j), 145.3, 145.1 (C-a and C-a'), 138.6 (C-b and C-b'), 132.8 (C-15 and C-g), 131.2 (C-7), 130.0, 129.9 (C-d and C-d'), 129.7, 129.6 (C-c and C-c'), 128.8 (C-17), 128.6 (C13 or C-14 and C-h), 127.7, 127.6 (C-2 and C-2'), 127.2, 127.1 (C-4 and C-4'), 126.9 (C13 or C-14), 126.8, 126.7 (C-5 and C-5'), 125.7 (C-1 or C-1'), 125.7(C-f), 125.6 (C-1 or C-1' and C-12), 124.7, 124.5 (C-3 and C-3'), 123.2, 123.1 (C-6 and C-6'), 121.1 (C-19), 116.9 (C-18), 116.2 (C-8), 81.8 (C-i), 60.2 (C-9), 58.8 (C-11), 41.6 (C-10), 26.0 (C-16); ESI-HRMS for $C_{47}H_{45}BN_3O_2$ $[M + H]^+$ calcd: 694.3607, found: 694.3610.

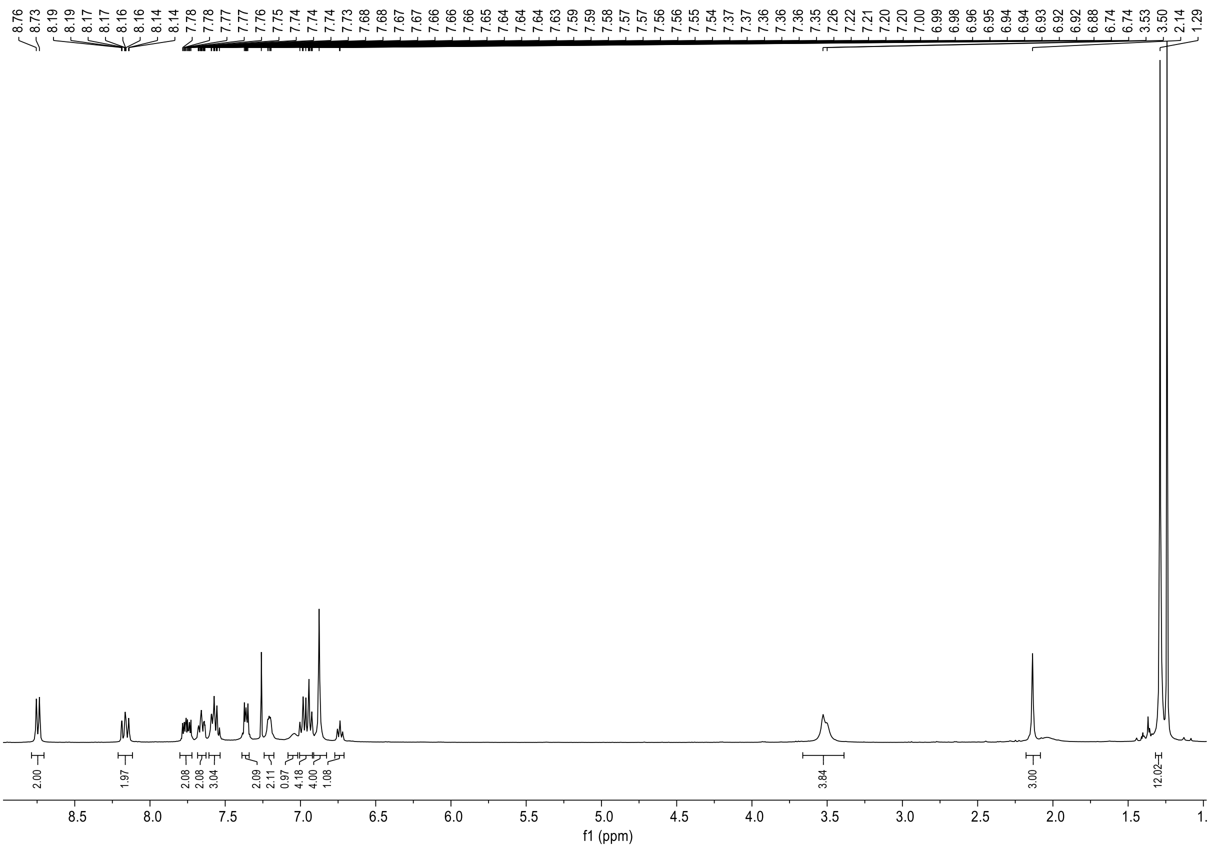

^{1}H NMR spectrum of compound **6** ($CDCl_3$, 400 MHz).

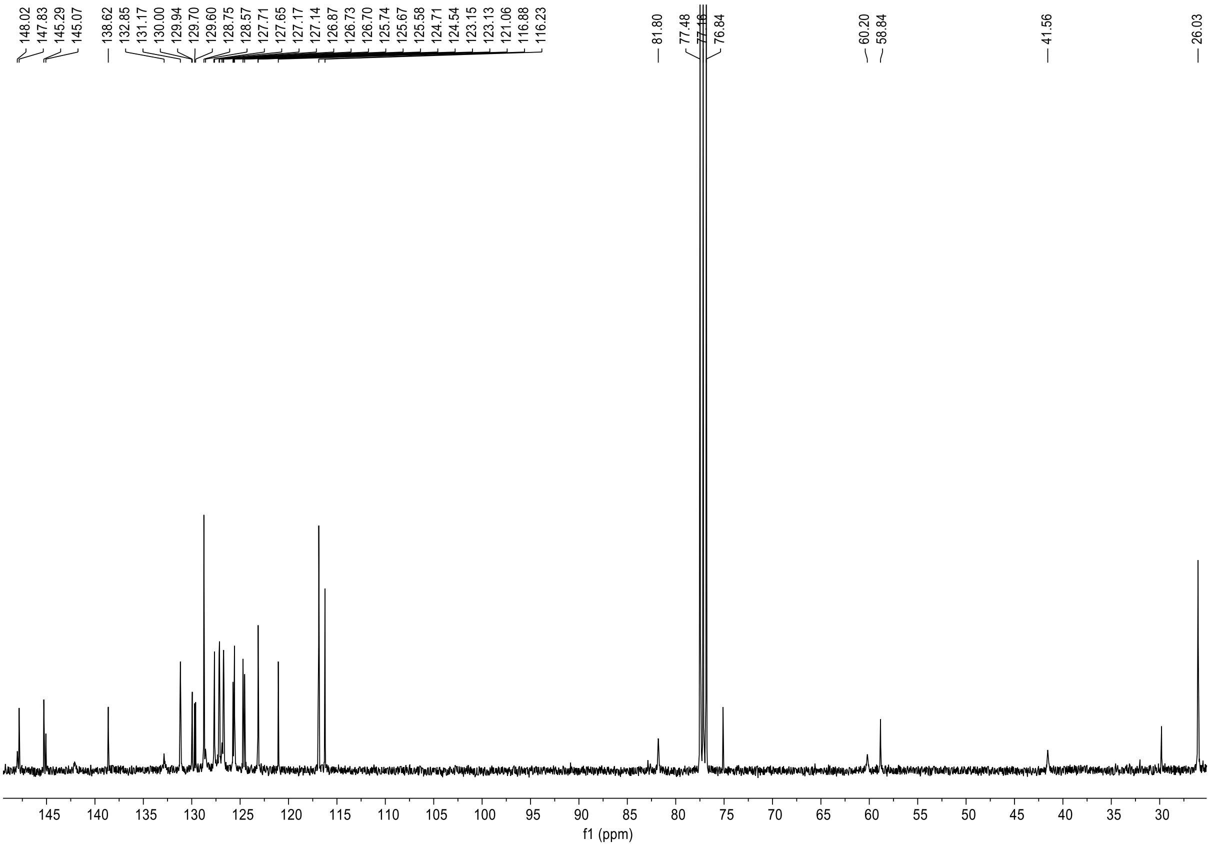

^{13}C NMR spectrum of compound **6** ($CDCl_3$, 100 MHz).

Synthesis of compound 2.

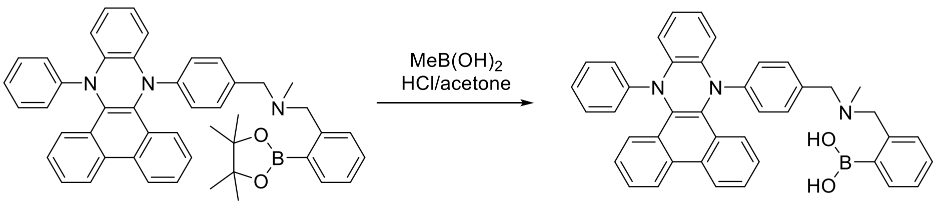

Into a small round-bottom flask, pinacol ester derivative **6** (100 mg, 0.14 mmol) and methylboronic acid (89 mg, 1.44 mmol) were added and dissolved in acetone/0.2 m HCl (4 mL, 1:1, v/v). The reaction mixture was stirred at room temperature overnight. Upon completion of the reaction as shown by TLC, the reaction mixture was concentrated to dryness, redissolved in $CHCl_3$ and dried *in vacuo*. The residue was purified by flash silica chromatography ($CHCl_3$/MeOH/TFA, 20:1:0 → 10:1:0.01) to afford compound **2** (83 mg, 94%) as a yellowish amorphous solid.

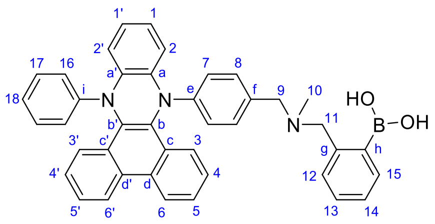

^{1}H NMR (400 MHz, MeOD-d_4) δ 8.85 (dd, J = 8.2, 5.5 Hz, 2H, H-6 and H-6'), 8.05 (t, J = 8.2 Hz, 2H, H-3 and H-3'), 7.87 (m, 1H, H-2 or H-2'), 7.81 (m, 1H, H-2 or H-2'), 7.74 – 7.64 (m, 3H, H-5, H-5' and H-15), 7.58 (m, 1H, H-4 or H-4'), 7.54 (m, 1H, H-4 or H-4'), 7.49 – 7.42 (m, 4H, H-1, H-1', H-13 and H-14), 7.35 (m, 1H, H-12), 7.12 (d, J = 8.4 Hz, 2H, H-7), 7.01 – 6.89 (m, 6H, H-8, H16 and H-17), 6.69 (m, 1H, H-12), 4.50 – 4.04 (m, 4H, H-9 and H-11), 2.44 (s, 3H, H-10); ^{13}C NMR (101 MHz, MeOD-d_4) δ 150.6 (C-e), 148.5 (C-i), 146.5, 145.3 (C-a and C-a'), 139.8, 138.7 (C-b and C-b'), 135.1 (C-h), 132.9 (C-12 and C-g), 132.7 (C-7), 131.2 (C-d or C-d'), 131.1 (C-13), 131.0 (C-d or C-d'), 130.2, 130.1 (C-c and C-c'), 130.0 (C-14), 129.6 (C-16), 128.9, 128.5 (C-2 and C-2'), 128.1 (C-4 or C-4'), 127.9 (C-5, C-5' and C-15), 127.8 (C-4 or C-4'), 127.2, 126.6 (C-1 and C-1'), 125.4, 124.8 (C-3 and C-3'), 124.3, 124.2 (C-6 and C-6'), 122.2 (C-18), 121.9 (C-f), 117.9 (C-17), 116.9 (C-8), 61.0 (C-9), 60.8 (C-11), 38.9 (C-10); ESI-HRMS for $C_{41}H_{35}BN_3O_2$ $[M + H]^+$ calcd: 612.2817, found: 612.2809.

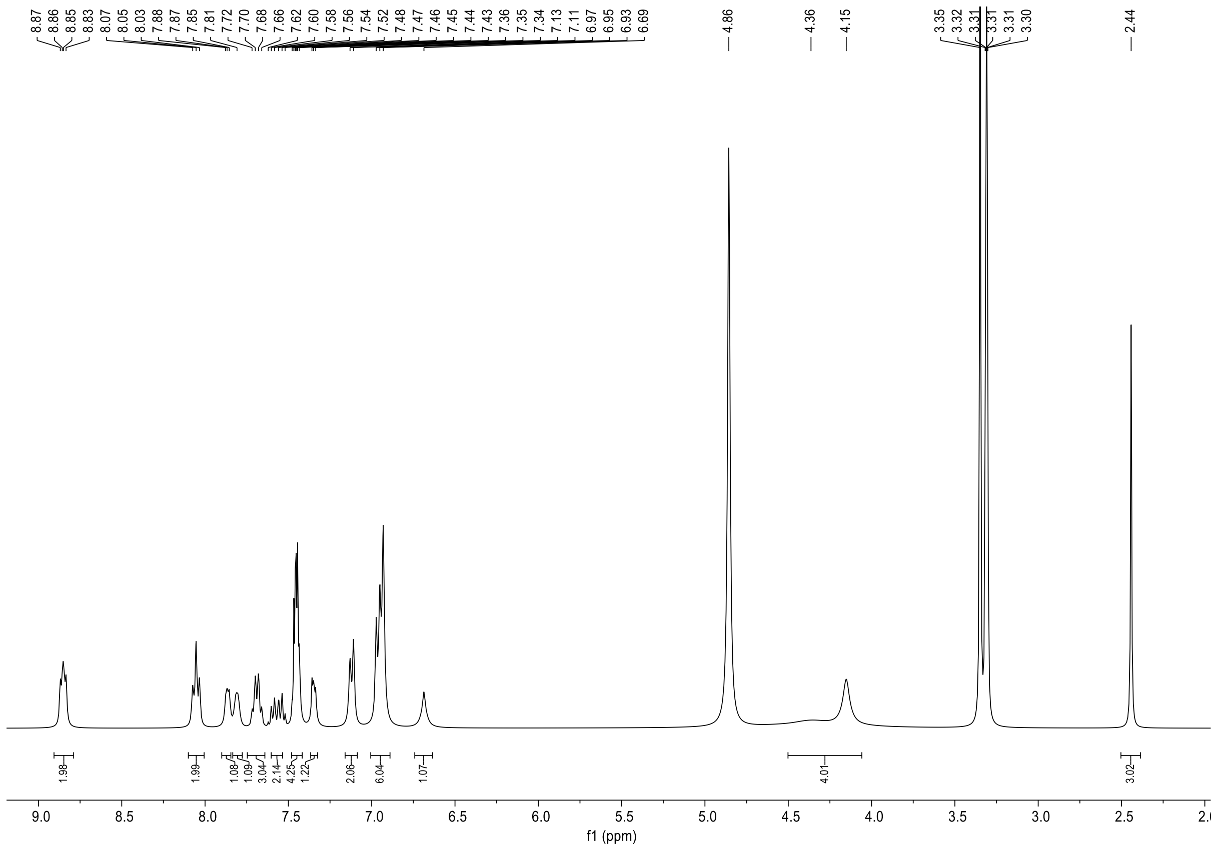

^{1}H NMR spectrum of compound **2** (CD_3OD, 400 MHz).

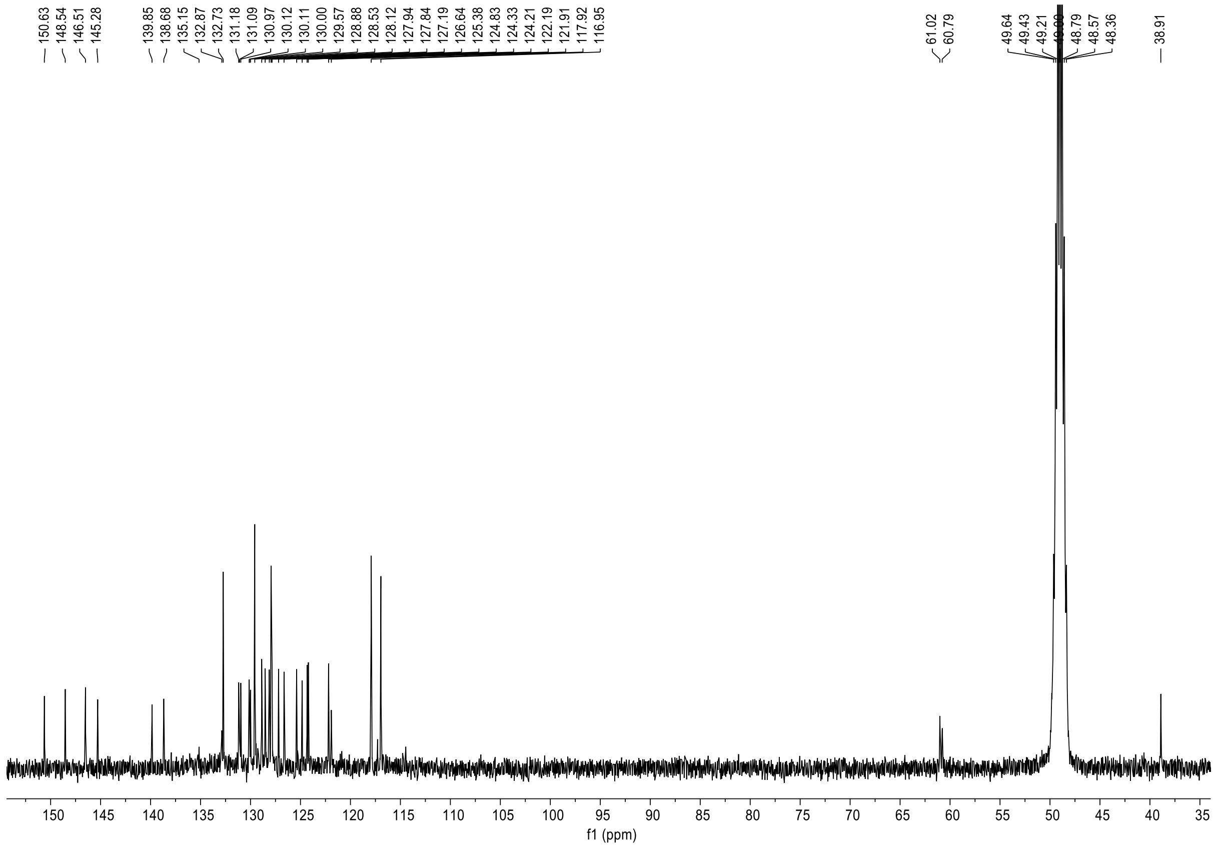

^{13}C NMR spectrum of compound **2** (CD_3OD, 100 MHz).

3. Fluorescent response of 1 to D-glucose, D-fructose and D-galactose.

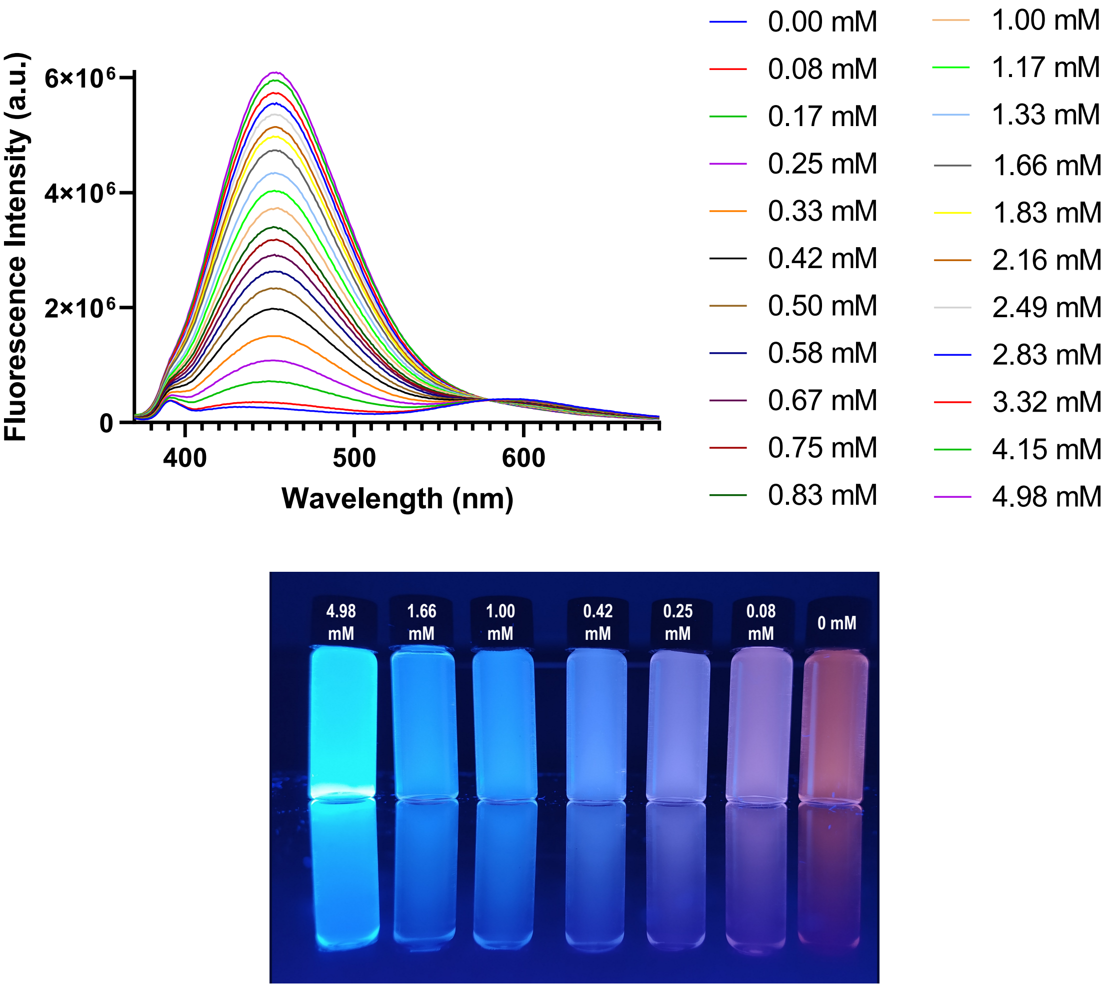

Figure S1. Fluorescence titration of **1** with D-glucose associated with the photographs showing the fluorescence features of **1** with various amounts of D-glucose under irradiation with UV light. λ_{ex} = 350 nm; [**1**] = 1 μM; solvent = MeOH/10 mM phosphate buffer (80:20).

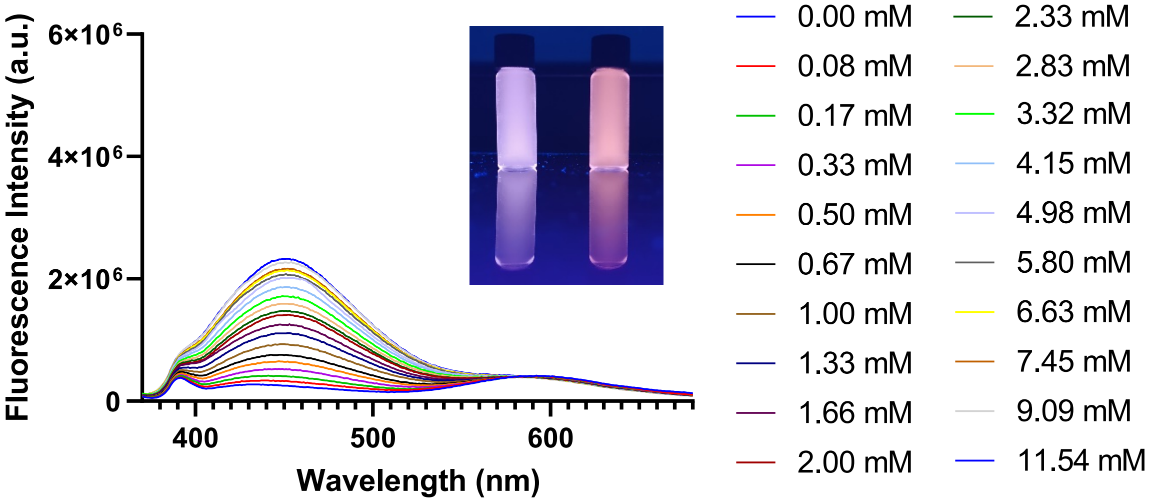

Figure S3. Fluorescence titration of **1** with D-galactose. λ_{ex} = 350 nm; [**1**] = 1 µM; solvent = MeOH/10 mM phosphate buffer (80:20). Inset: Fluorescence image of **1**⊃D-galactose (left) and **1** (right) recorded upon addition with 365 nm UV light.

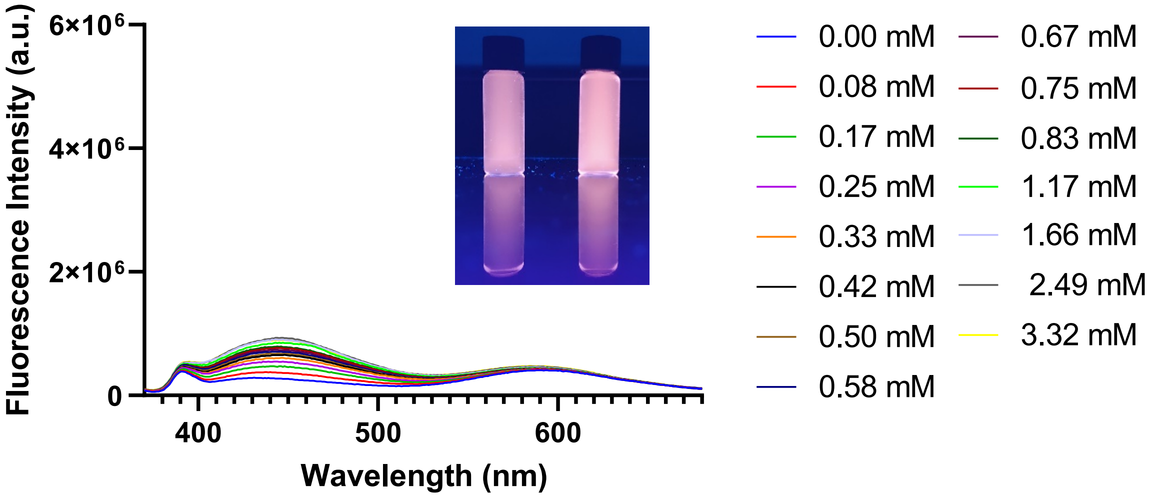

Figure S3. Fluorescence titration of **1** with D-fructose. λ_{ex} = 350 nm; [**1**] = 1 µM; solvent = MeOH/10 mM phosphate buffer (80:20). Inset: Fluorescence image of **1**⊃D-fructose (left) and **1** (right) recorded upon addition with 365 nm UV light.

4. Fluorescent response of 1 to other monosaccharides.

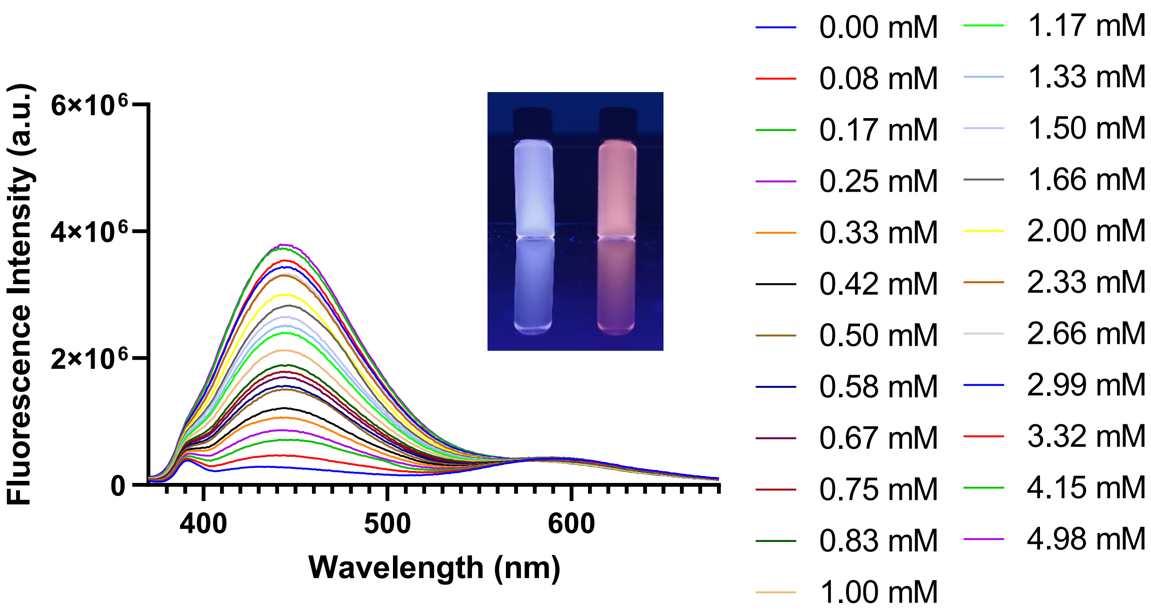

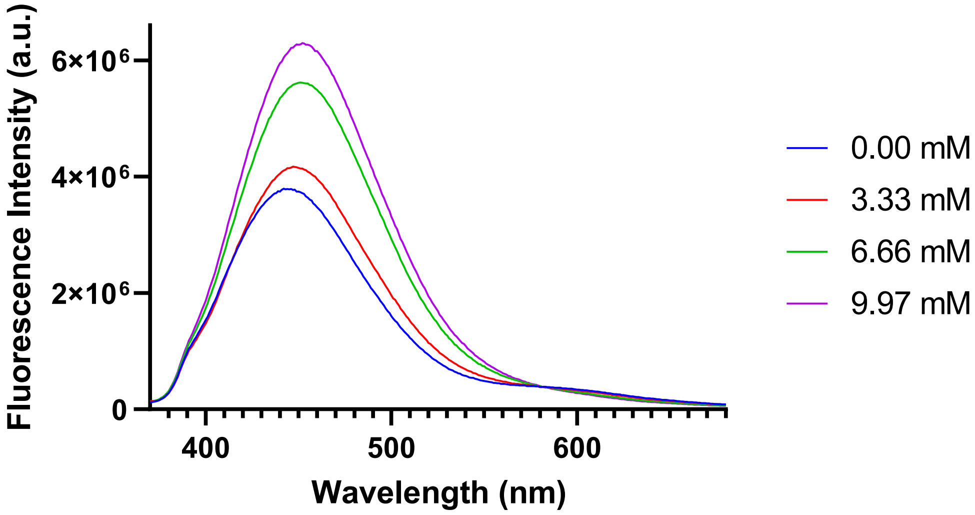

Figure S4. Fluorescence titrations of **1** with D-xylose (top) and **1**⊃D-xylose (4.98 mM) with D-glucose (bottom). λ_{ex} = 350 nm; [**1**] = 1 μM; solvent = MeOH/10 mM phosphate buffer (80:20). Inset: Fluorescence image of **1**⊃D-xylose (left) and **1** (right) recorded upon addition with 365 nm UV light.

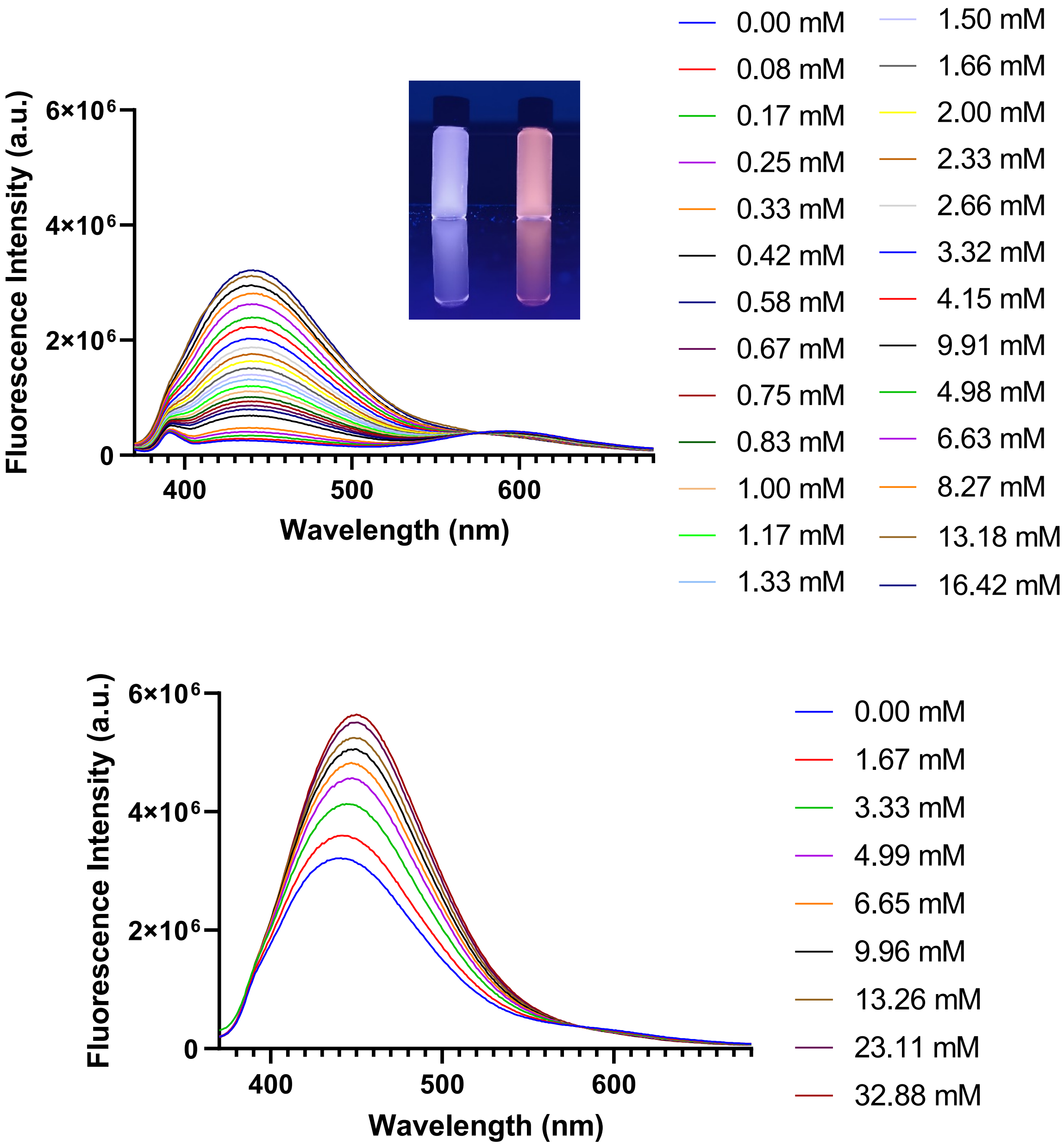

Figure S5. Fluorescence titrations of **1** with D-mannose (top) and **1**⊃D-mannose (16.42 mM) with D-glucose (bottom). λ_{ex} = 350 nm; [**1**] = 1 μM; solvent = MeOH/10 mM phosphate buffer (80:20). Inset: Fluorescence image of **1**⊃D-mannose (left) and **1** (right) recorded upon addition with 365 nm UV light.

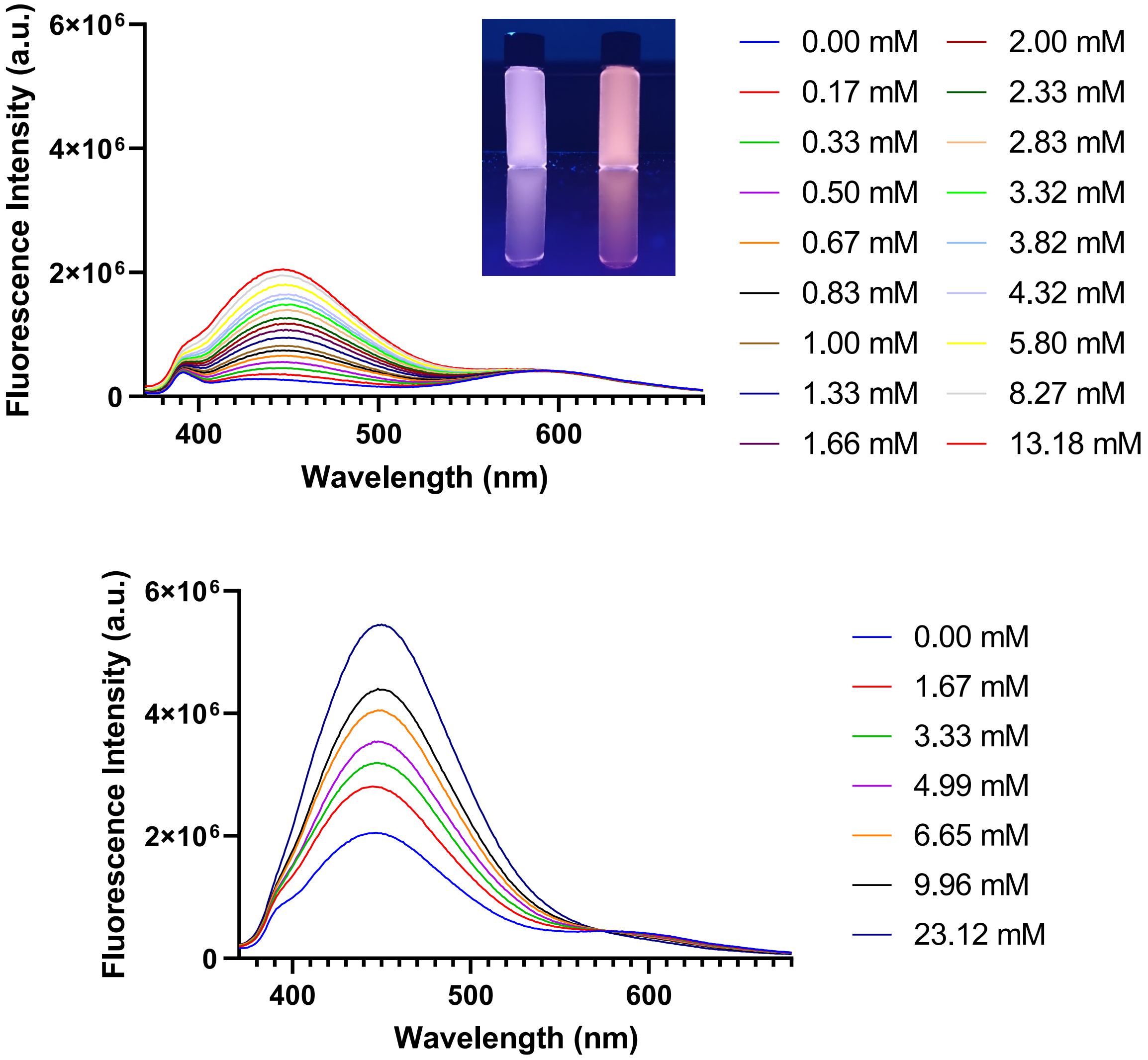

Figure S6. Fluorescence titrations of **1** with L-arabinose (top) and **1**⊃L-arabinose (13.18 mM) with D-glucose (bottom). λ_{ex} = 350 nm; [**1**] = 1 μM; solvent = MeOH/10 mM phosphate buffer (80:20). Inset: Fluorescence image of **1**⊃L-arabinose (left) and **1** (right) recorded upon addition with 365 nm UV light.

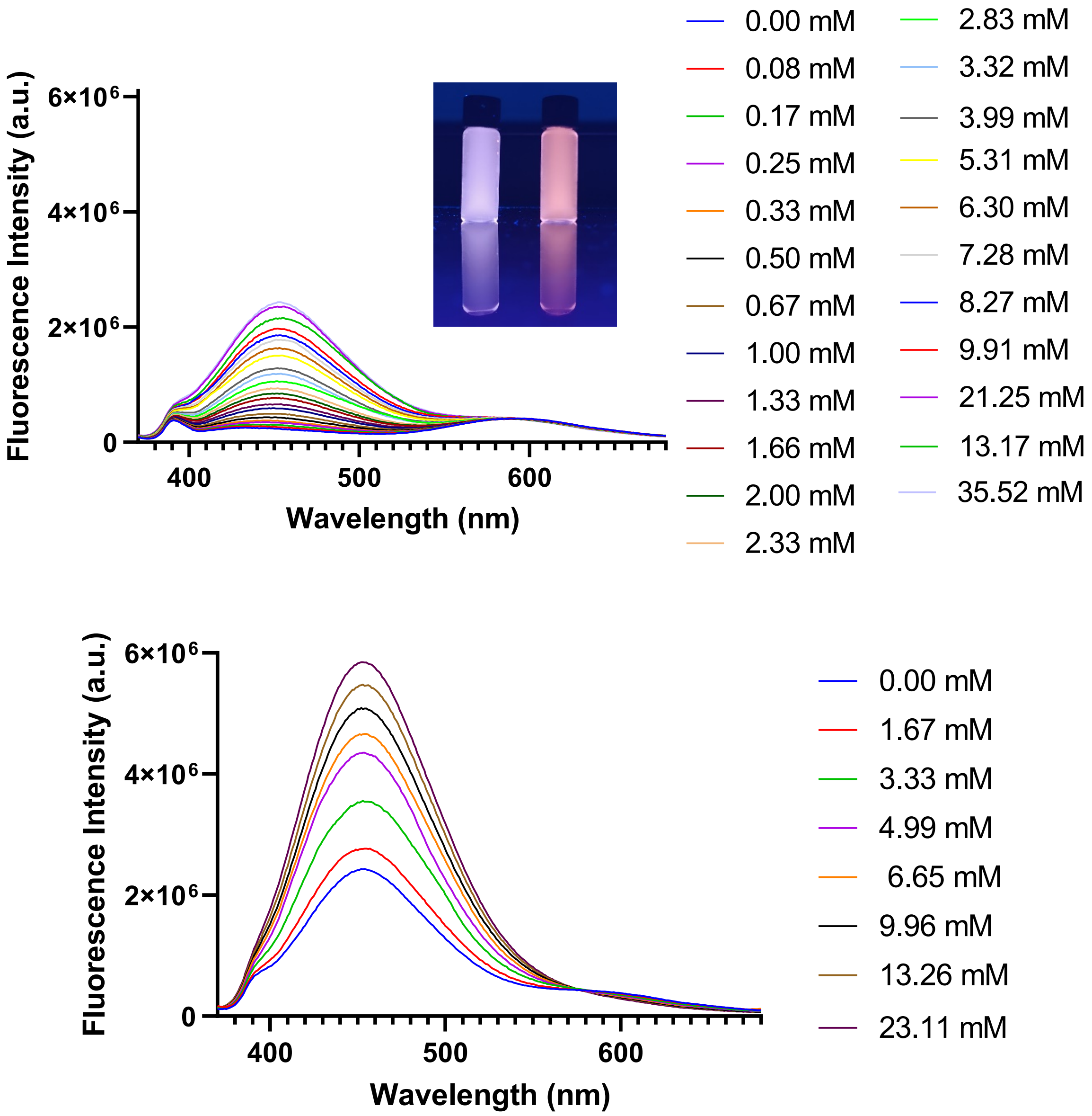

Figure S7. Fluorescence titrations of **1** with L-fucose (top) and **1**⊃L-fucose (35.52 mM) with D-glucose (bottom). λ_{ex} = 350 nm; [**1**] = 1 μM; solvent = MeOH/10 mM phosphate buffer (80:20). Inset: Fluorescence image of **1**⊃L-fucose (left) and **1** (right) recorded upon addition with 365 nm UV light.

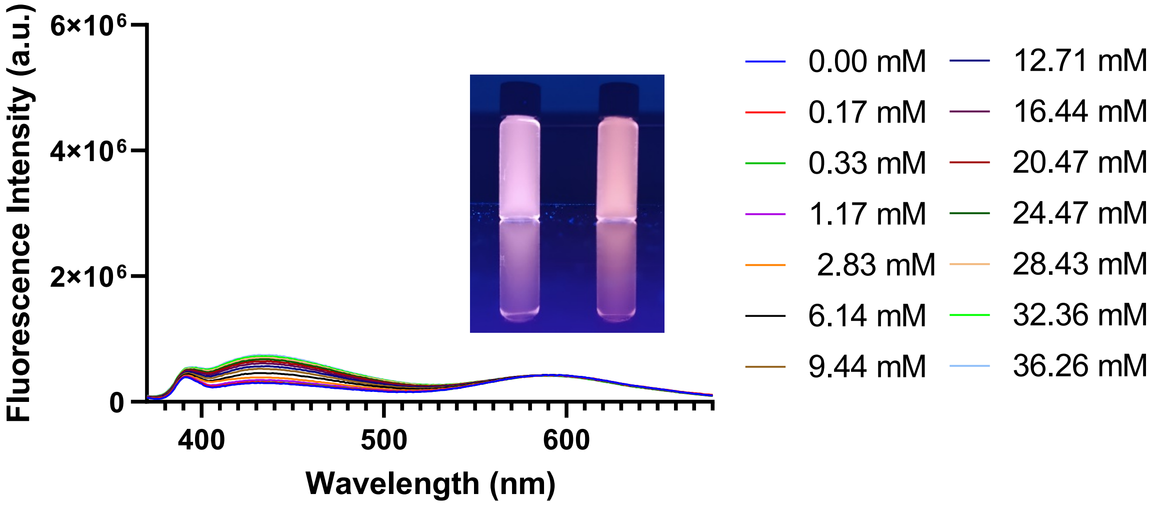

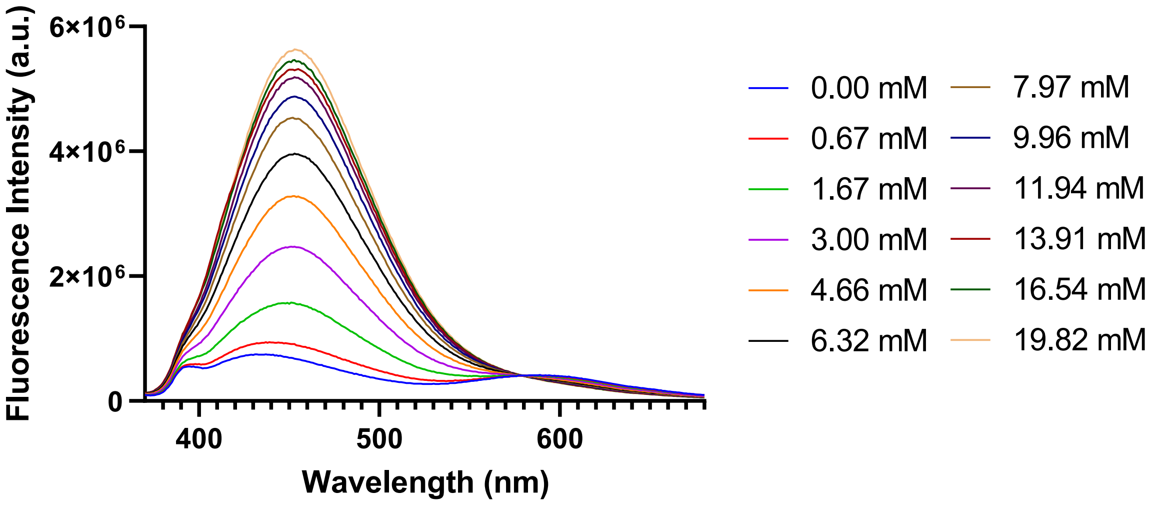

Figure S8. Fluorescence titrations of **1** with L-rhamnose (top) and **1**⊃L-rhamnose (36.26 mM) with D-glucose (bottom). λ_{ex} = 350 nm; [**1**] = 1 μM; solvent = MeOH/10 mM phosphate buffer (80:20). Inset: Fluorescence image of **1**⊃L-rhamnose (left) and **1** (right) recorded upon addition with 365 nm UV light.

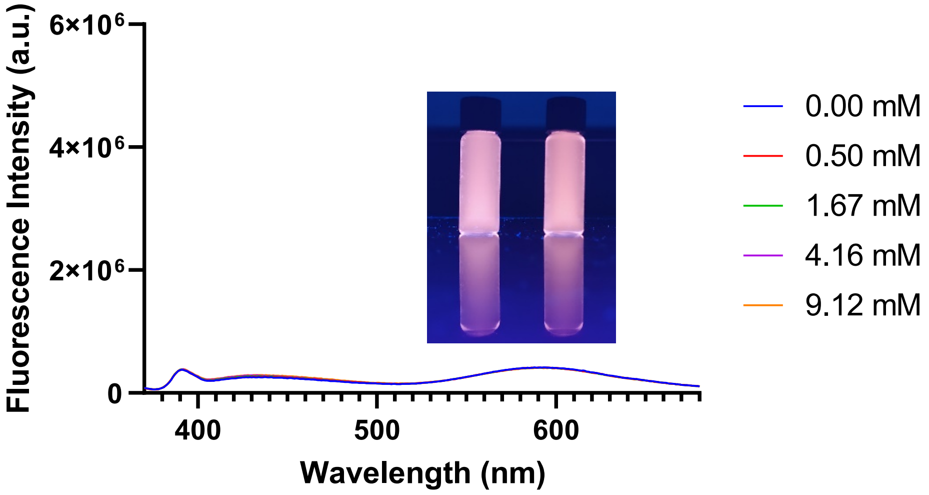

Figure S9. Fluorescence titration of **1** with methyl-β-D-glucopyranoside. λ_{ex} = 350 nm; [**1**] = 1 µM; solvent = MeOH/10 mM phosphate buffer (80:20). Inset: Fluorescence image of **1**⊃methyl-β-D-glucopyranoside (left) and **1** (right) recorded upon addition with 365 nm UV light.

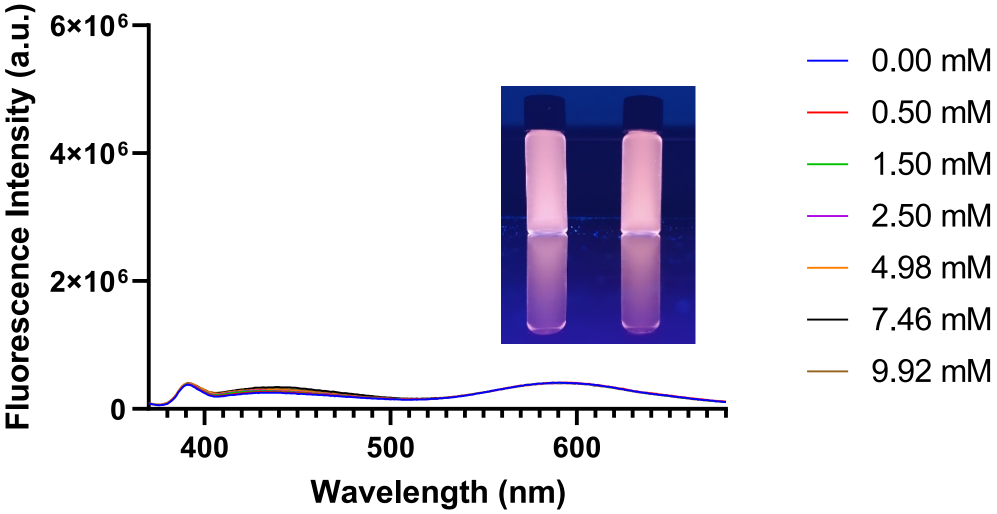

Figure S10. Fluorescence titration of **1** with *N*-acetyl-β-D-glucosamine. λ_{ex} = 350 nm; [**1**] = 1 µM; solvent = MeOH/10 mM phosphate buffer (80:20). Inset: Fluorescence image of **1**⊃*N*-acetyl-β-D-glucosamine (left) and **1** (right) recorded upon addition with 365 nm UV light.

5. Fluorescent response of 2 to monosaccharides.

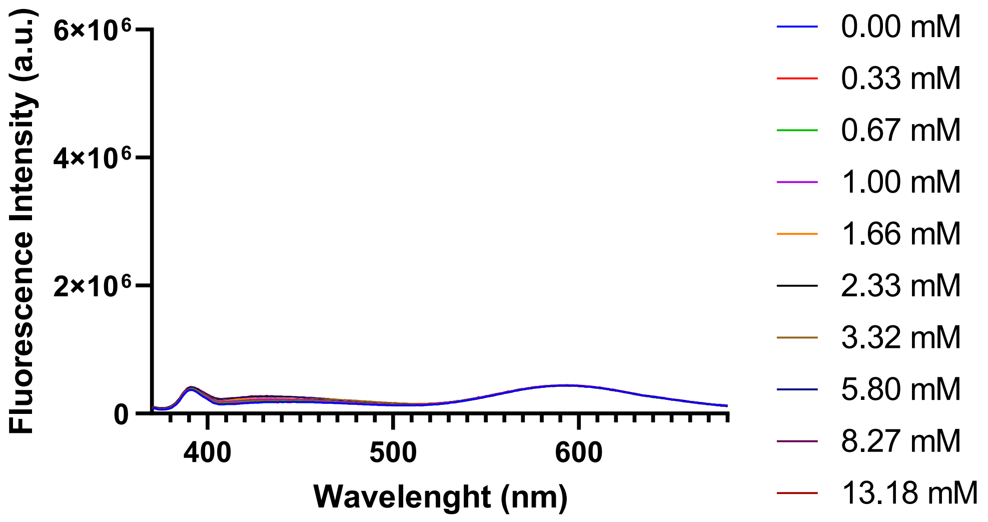

Figure S11. Fluorescence titration of **2** with L-arabinose. λ_{ex} = 350 nm; [**2**] = 1 μM; solvent = MeOH/10 mM phosphate buffer (80:20).

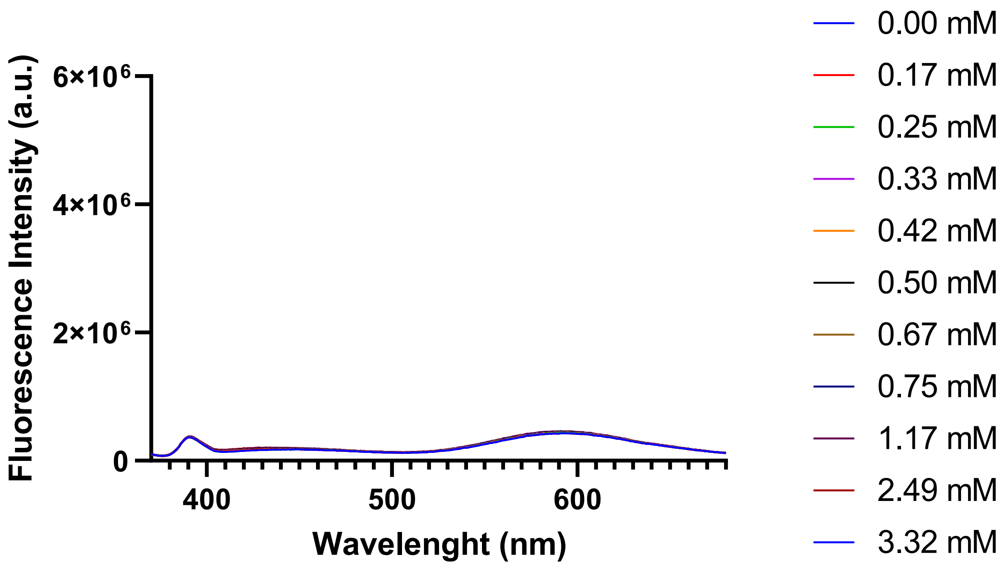

Figure S12. Fluorescence titration of **2** with D-fructose. λ_{ex} = 350 nm; [**2**] = 1 μM; solvent = MeOH/10 mM phosphate buffer (80:20).

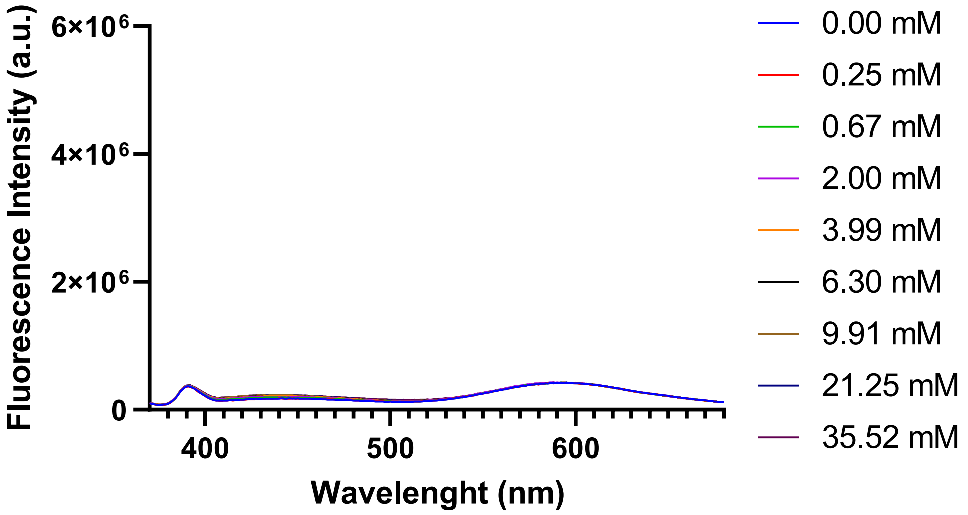

Figure S13. Fluorescence titration of **2** with L-fucose. λ_{ex} = 350 nm; [**2**] = 1 µM; solvent = MeOH/10 mM phosphate buffer (80:20).

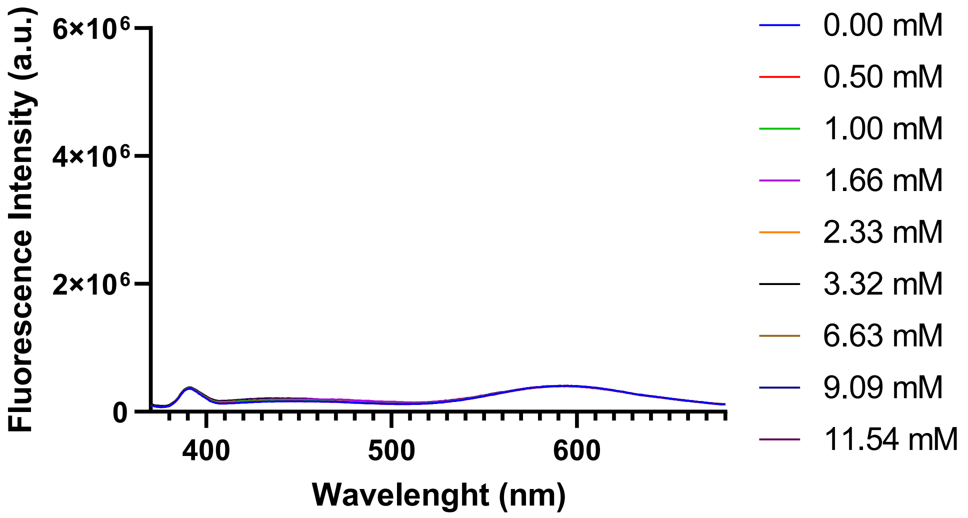

Figure S14. Fluorescence titration of **2** with D-galactose. λ_{ex} = 350 nm; [**2**] = 1 µM; solvent = MeOH/10 mM phosphate buffer (80:20).

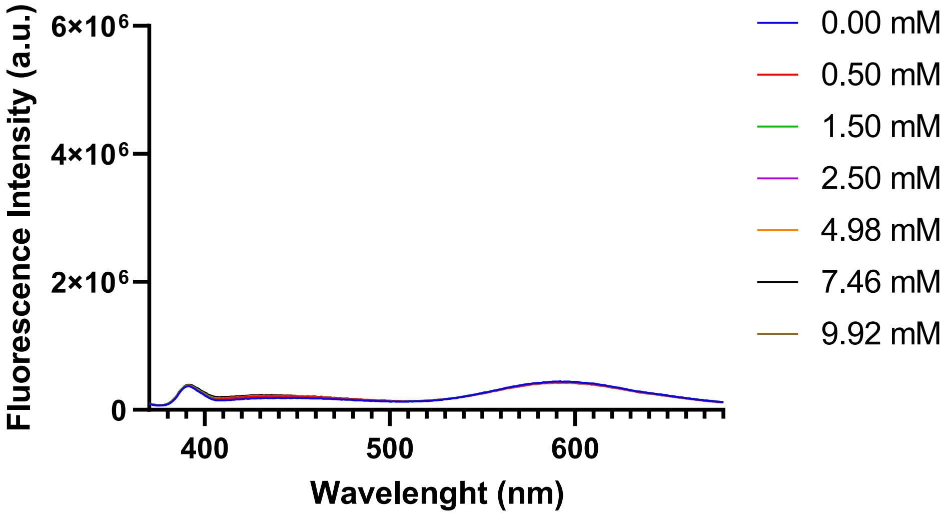

Figure S15. Fluorescence titration of **2** with *N*-acetyl-β-D-glucosamine. λ_{ex} = 350 nm; [**2**] = 1 μM; solvent = MeOH/10 mM phosphate buffer (80:20).

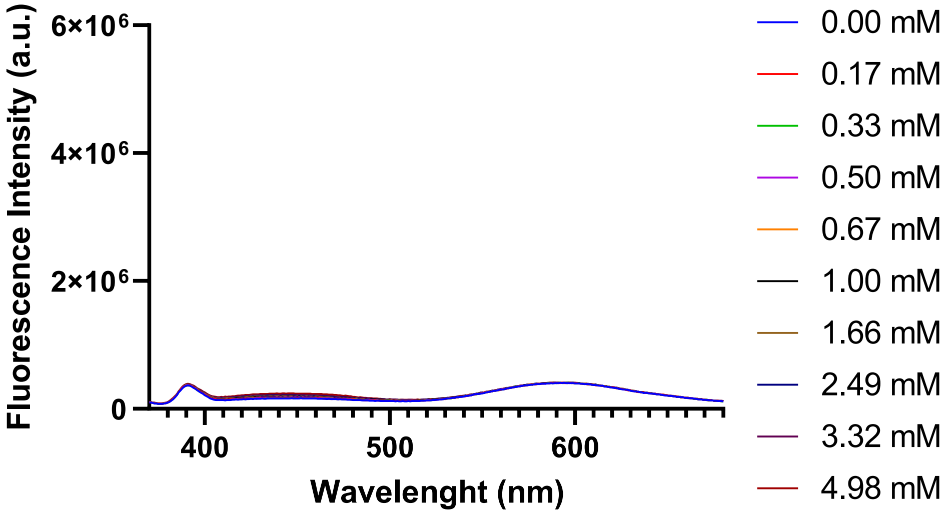

Figure S16. Fluorescence titration of **2** with D-glucose. λ_{ex} = 350 nm; [**2**] = 1 μM; solvent = MeOH/10 mM phosphate buffer (80:20)

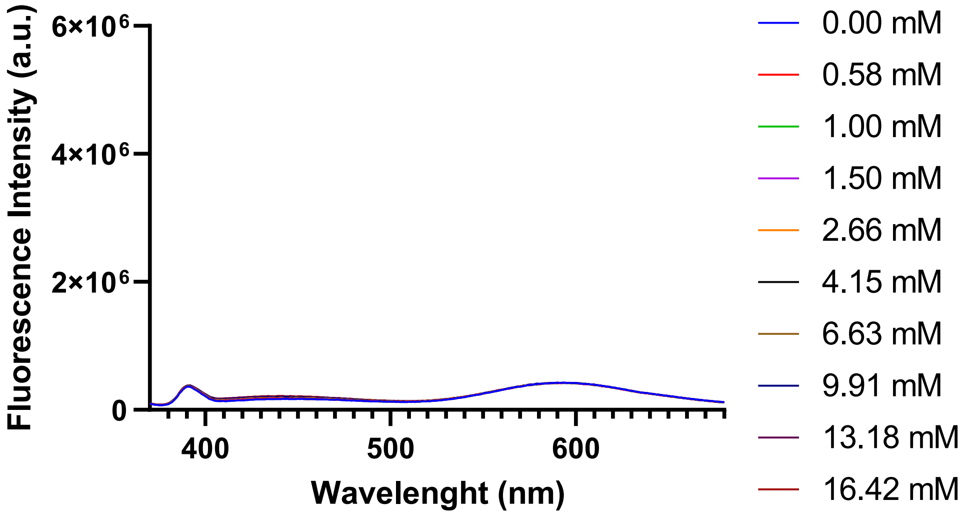

Figure S17. Fluorescence titration of **2** with D-mannose. λ_{ex} = 350 nm; [**2**] = 1 µM; solvent = MeOH/10 mM phosphate buffer (80:20).

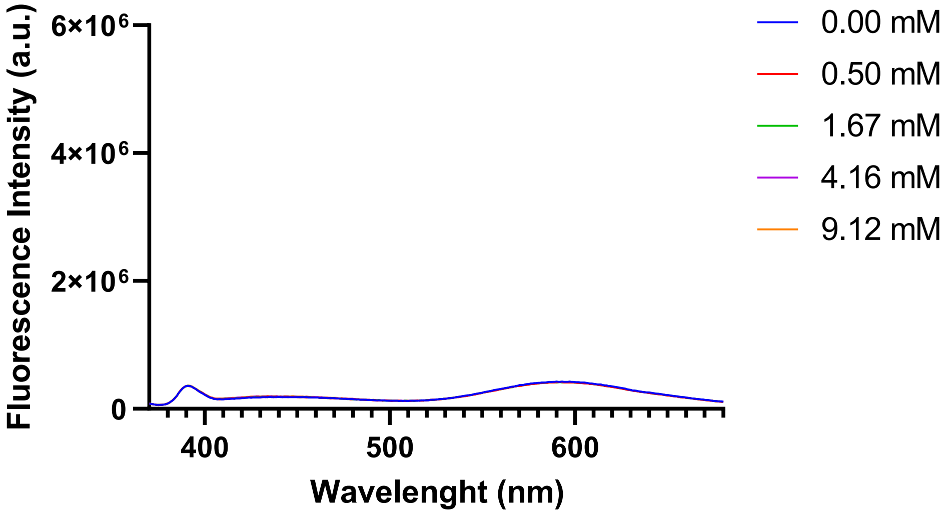

Figure S18. Fluorescence titration of **2** with methyl-β-D-glucopyranoside. λ_{ex} = 350 nm; [**2**] = 1 µM; solvent = MeOH/10 mM phosphate buffer (80:20).

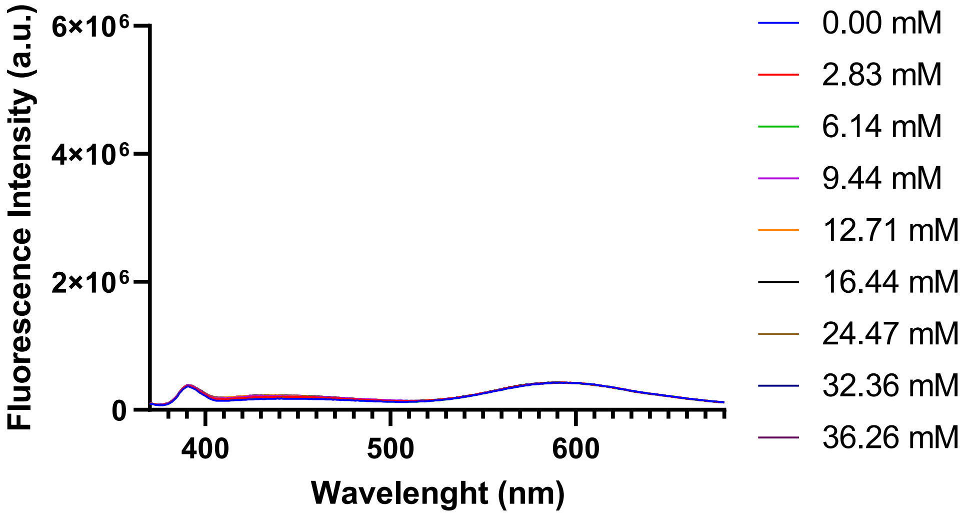

Figure S19. Fluorescence titration of **2** with L-rhamnose. λ_{ex} = 350 nm; [**2**] = 1 µM; solvent = MeOH/10 mM phosphate buffer (80:20).

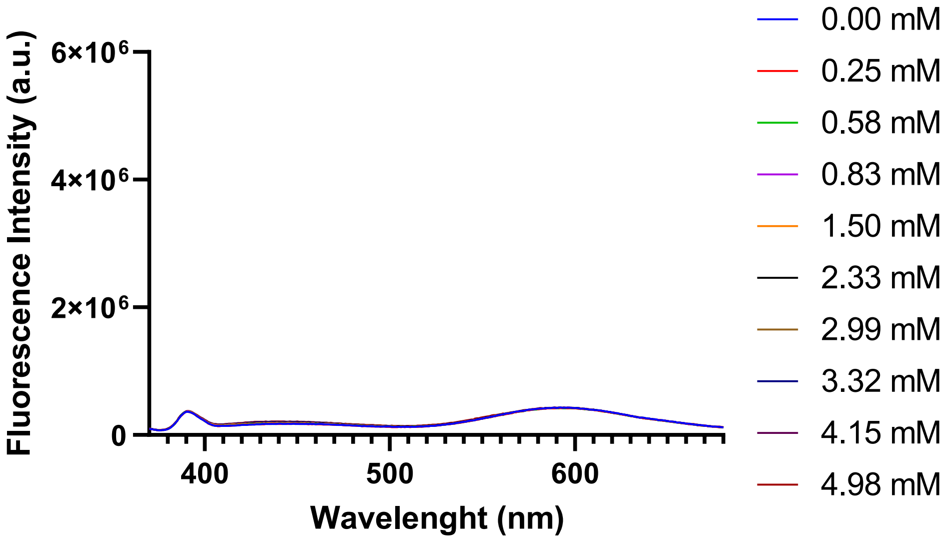

Figure S20. Fluorescence titration of **2** with D-xylose. λ_{ex} = 350 nm; [**2**] = 1 µM; solvent = MeOH/10 mM phosphate buffer (80:20).

6. Titrations Fitting and Binding Isotherms

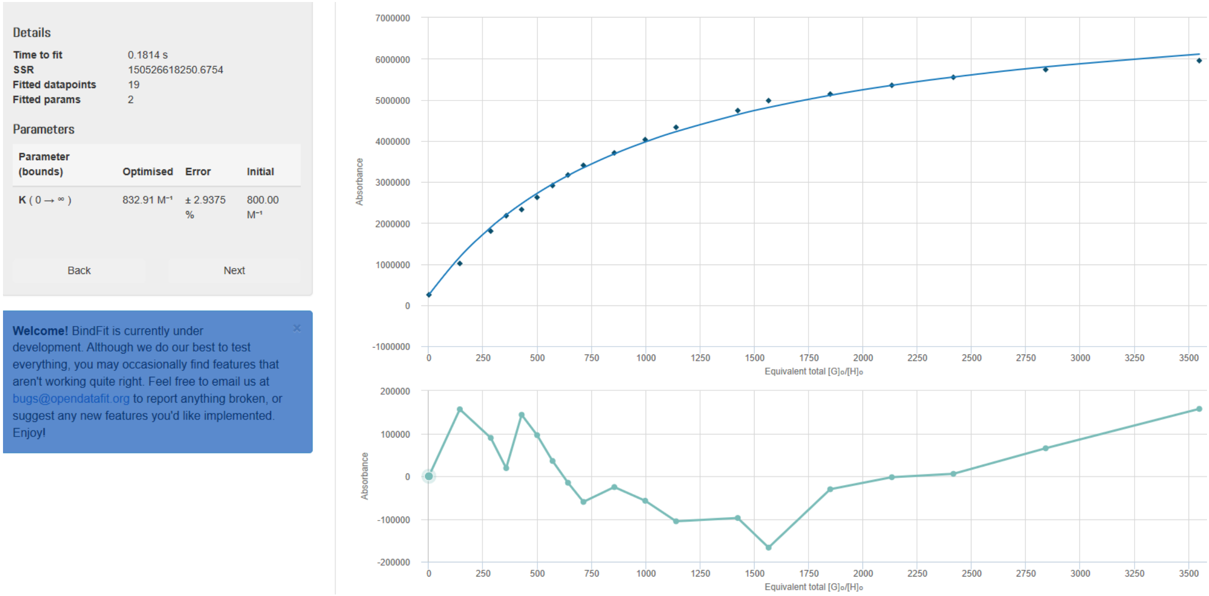

Figure S21. Least-squares nonlinear fitting of the normalized change in fluorescence at 452 nm obtained as a function of concentration on the basis of the fluorescence titration of **1** with D-glucose. The solid lines were obtained from nonlinear curve-fitting to a 1:1 binding model using the www.supramolecular.org web applet. (http://app.supramolecular.org/bindfit/view/b2013745-77f6-49d2-8fcd-b8fd73fa64c8)

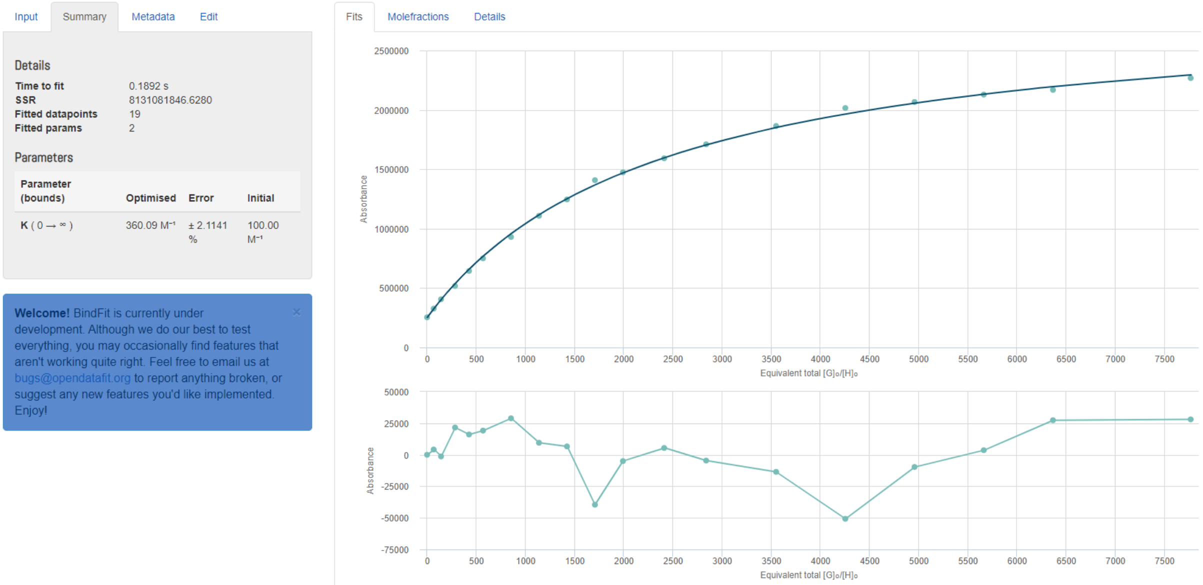

Figure S22. Least-squares nonlinear fitting of the normalized change in fluorescence at 450 nm obtained as a function of concentration on the basis of the fluorescence titration of **1** with D-galactose. The solid lines were obtained from nonlinear curve-fitting to a 1:1 binding model using the www.supramolecular.org web applet. (http://app.supramolecular.org/bindfit/view/23d09524-6676-4a69-b4a0-ef9258f99f2d)

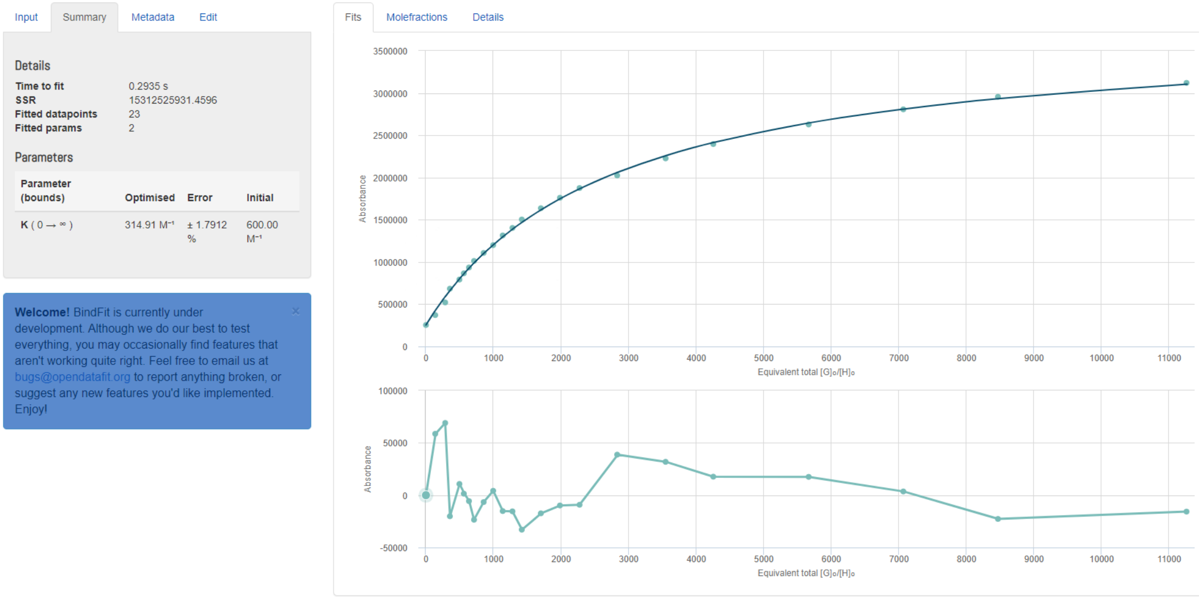

Figure S23. Least-squares nonlinear fitting of the normalized change in fluorescence at 440 nm obtained as a function of concentration on the basis of the fluorescence titration of **1** with D-mannose. The solid lines were obtained from nonlinear curve-fitting to a 1:1 binding model using the www.supramolecular.org web applet. (http://app.supramolecular.org/bindfit/view/aeda20fb-7264-4b2c-bf2d-92f47fa27aa0)

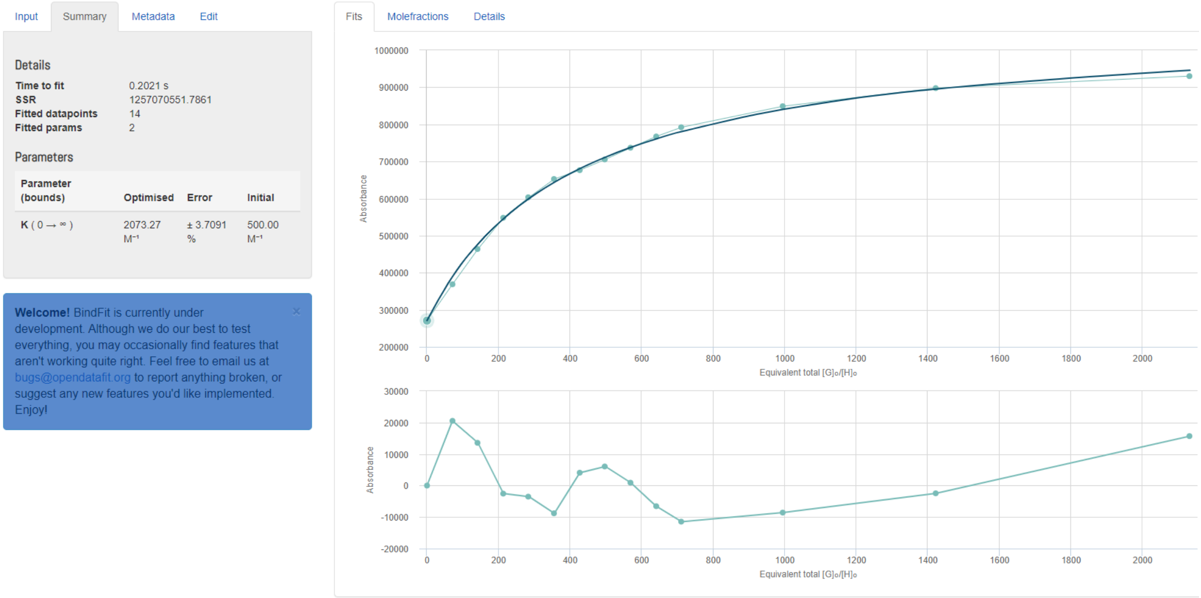

Figure S24. Least-squares nonlinear fitting of the normalized change in fluorescence at 444 nm obtained as a function of concentration on the basis of the fluorescence titration of **1** with D-fructose. The solid lines were obtained from nonlinear curve-fitting to a 1:1 binding model using the www.supramolecular.org web applet. (http://app.supramolecular.org/bindfit/view/92c0c0b6-6006-4cea-95b7-228c0ecd0c98)

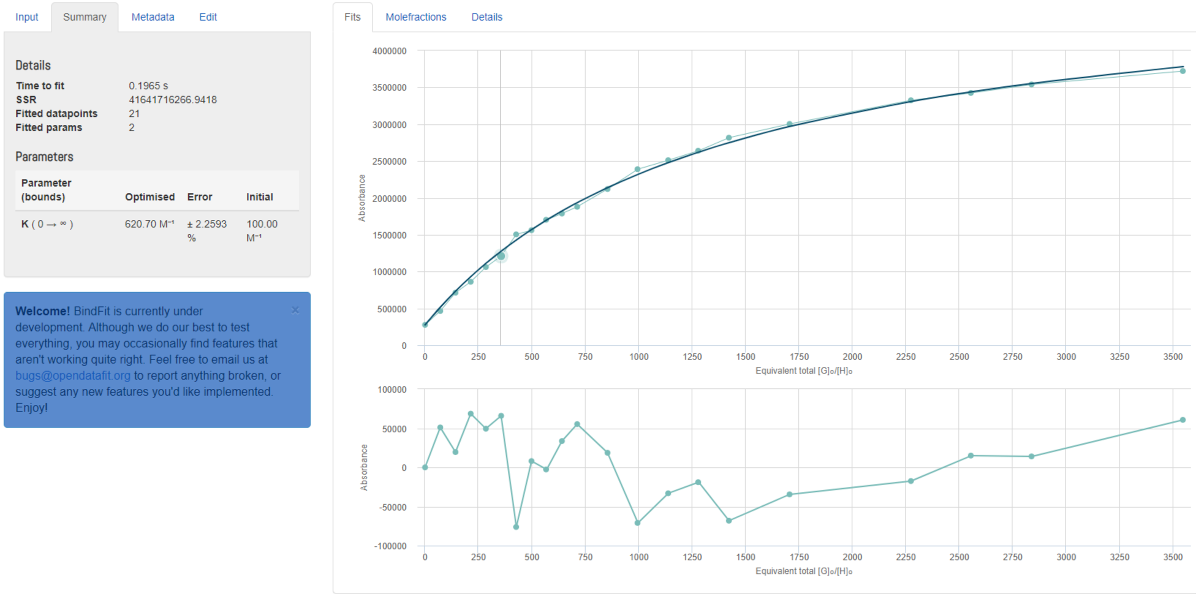

Figure S25. Least-squares nonlinear fitting of the normalized change in fluorescence at 444 nm obtained as a function of concentration on the basis of the fluorescence titration of **1** with D-xylose. The solid lines were obtained from nonlinear curve-fitting to a 1:1 binding model using the www.supramolecular.org web applet. (http://app.supramolecular.org/bindfit/view/b727c83f-eacf-4239-b86a-9be10da7b2df)

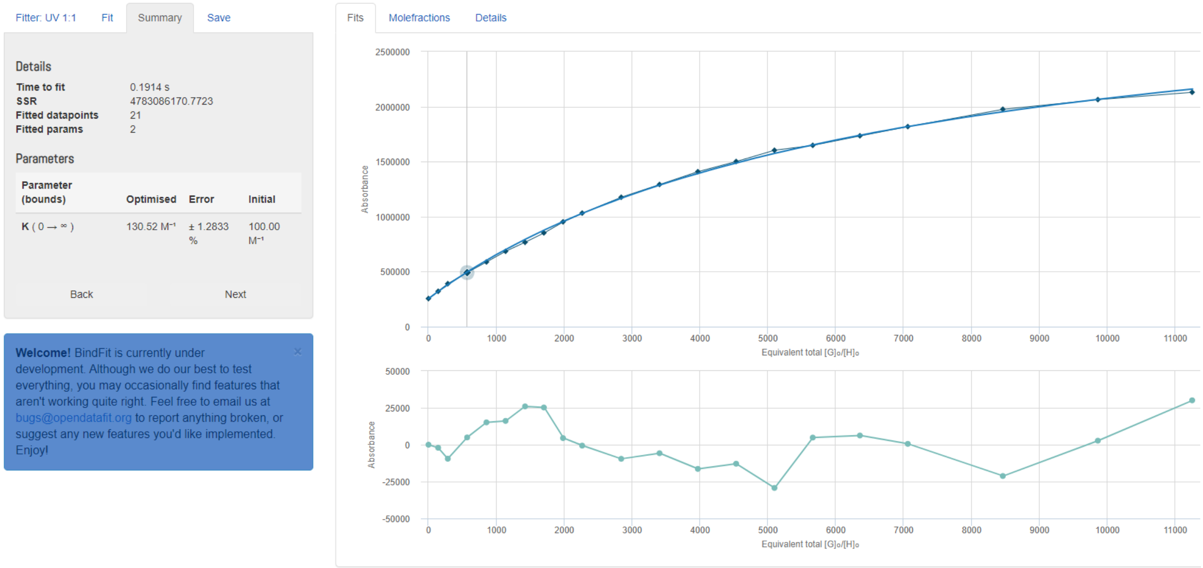

Figure S26. Least-squares nonlinear fitting of the normalized change in fluorescence at 453 nm obtained as a function of concentration on the basis of the fluorescence titration of **1** with L-fucose. The solid lines were obtained from nonlinear curve-fitting to a 1:1 binding model using the www.supramolecular.org web applet. (http://app.supramolecular.org/bindfit/view/076caeb1-1fc7-4cff-bc72-53539638da10)

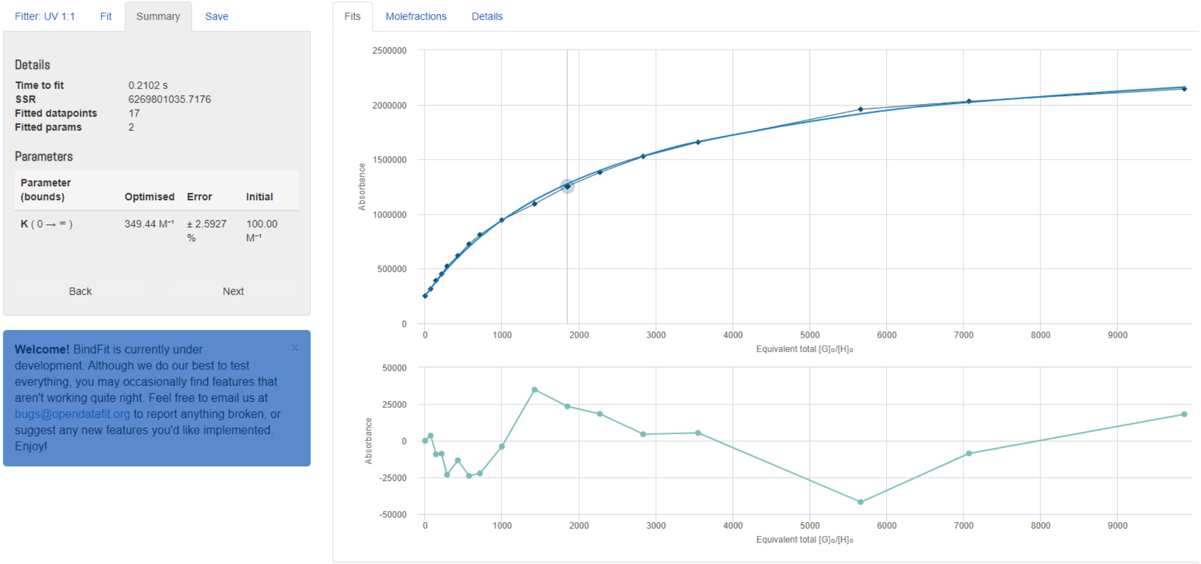

Figure S27. Least-squares nonlinear fitting of the normalized change in fluorescence at 447 nm obtained as a function of concentration on the basis of the fluorescence titration of **1** with L-arabinose. The solid lines were obtained from nonlinear curve-fitting to a 1:1 binding model using the www.supramolecular.org web applet. (http://app.supramolecular.org/bindfit/view/8b24bdf0-74e6-481d-bfe1-d2c7daf4dce7)

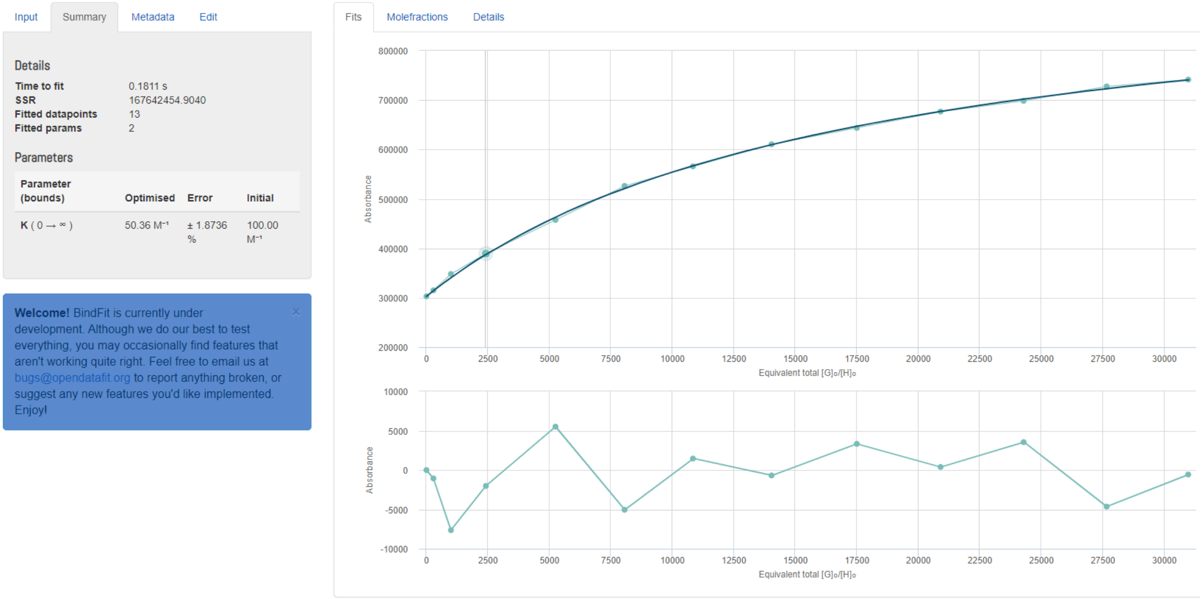

Figure S28. Least-squares nonlinear fitting of the normalized change in fluorescence at 433 nm obtained as a function of concentration on the basis of the fluorescence titration of **1** with L-rhamnose. The solid lines were obtained from nonlinear curve-fitting to a 1:1 binding model using the www.supramolecular.org web applet. (http://app.supramolecular.org/bindfit/view/da11d50f-21f2-4232-8d0e-0062f4543c75)

7. MS spectrum of complex 1⊃D-glucose.

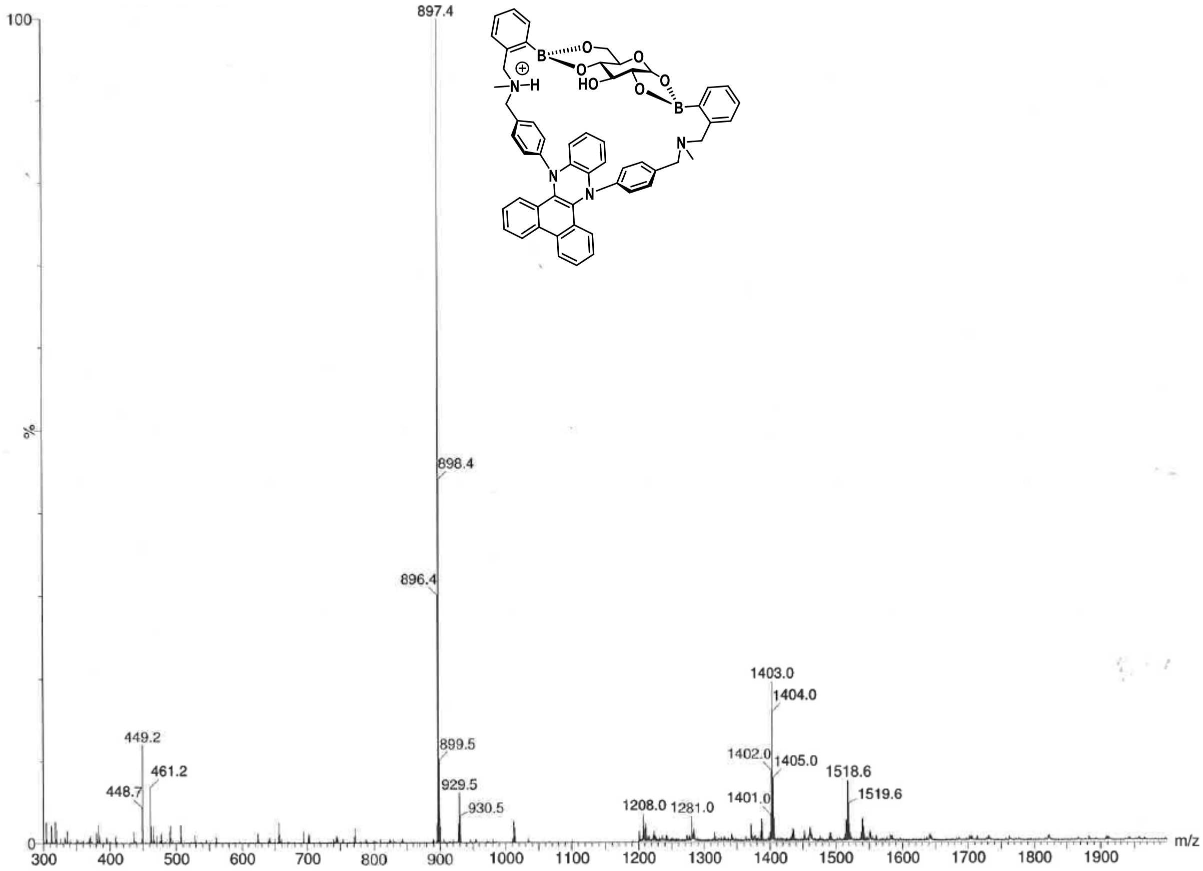

Figure S29. Nanospray spectrum for complex **1**⊃D-glucose.

8. Determination of the limit of detection (LOD).

The limit of detection (LOD) was calculated from the equation:

$$\text{Limit of Dectection (LOD)} = 3\sigma/k$$

being σ the standard deviation of emission intensity for the initial host solution (c_0 = 1 μM in 3.0 mL of MeOH/10 mM phosphate buffer (pH 7.4) (80:20)); k is the slope of the calibration curve (Figures S30–S37). The standard deviation for the emission intensity of **1** was obtained by measuring 5 times a solution of free receptor.

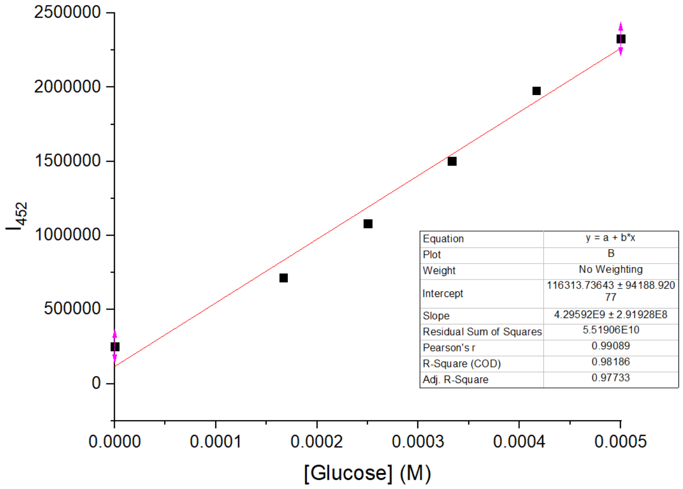

Figure S30. Calibration curve for LOD determination: plot of the fluorescence intensity of receptor **1** versus the glucose concentration in MeOH/10 mM phosphate buffer (80:20); λ_{ex}: 350 nm; λ_{em}: 452 nm; $k = 4.29 \times 10^9$ M^{-1}; σ = 13538.81.

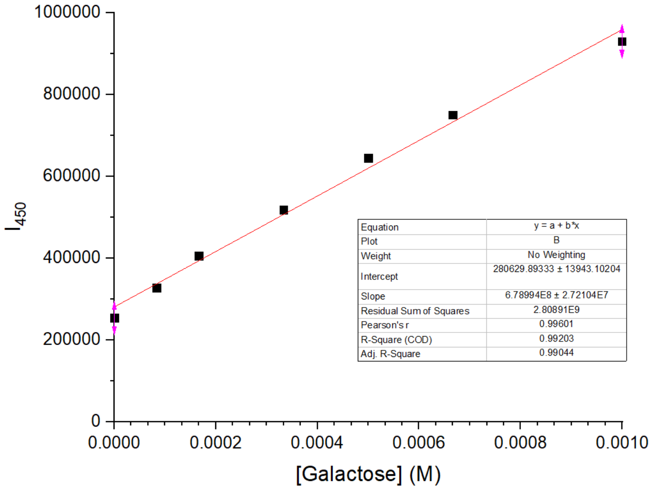

Figure S31. Calibration curve for LOD determination: plot of the fluorescence intensity of receptor **1** versus the galactose concentration in MeOH/10 mM phosphate buffer (80:20); λ_{ex}: 350 nm; λ_{em}: 450 nm; $k = 6.78 \times 10^8$ M^{-1}; σ = 14294.66.

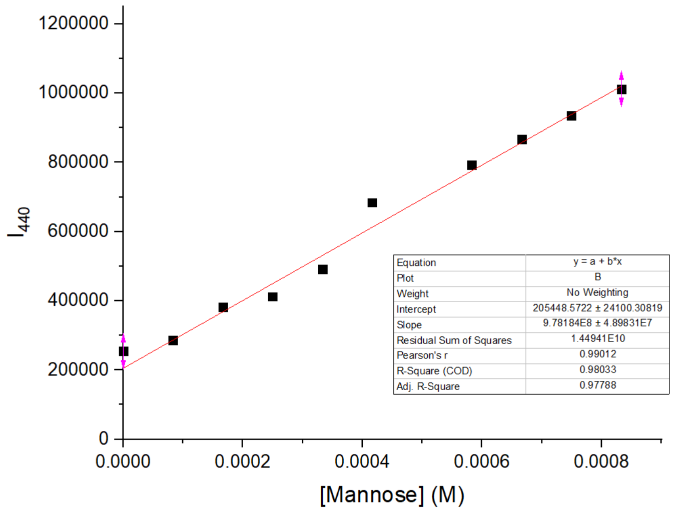

Figure S32. Calibration curve for LOD determination: plot of the fluorescence intensity of receptor **1** versus the mannose concentration in MeOH/10 mM phosphate buffer (80:20); λ_{ex}: 350 nm; λ_{em}: 440 nm; $k = 9.78 \times 10^8$ M^{-1}; σ = 14880.42.

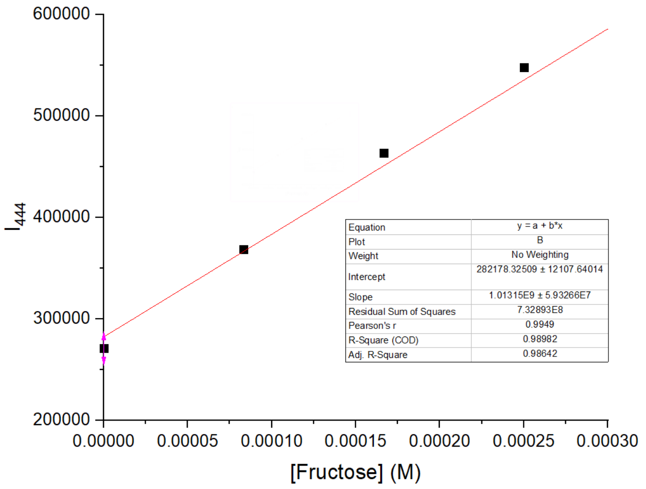

Figure S33. Calibration curve for LOD determination: plot of the fluorescence intensity of receptor **1** versus the fructose concentration in MeOH/10 mM phosphate buffer (80:20); λ_{ex}: 350 nm; λ_{em}: 444 nm; $k = 1.01 \times 10^9$ M^{-1}; σ = 13613.81.

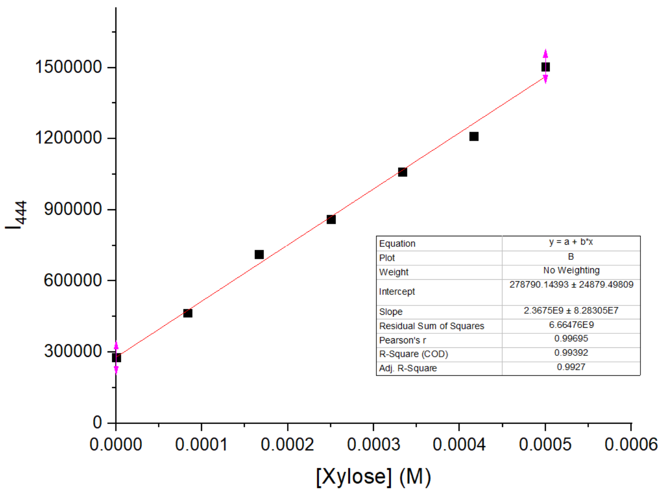

Figure S34. Calibration curve for LOD determination: plot of the fluorescence intensity of receptor **1** versus the xylose concentration in MeOH/10 mM phosphate buffer (80:20); λ_{ex}: 350 nm; λ_{em}: 444 nm; $k = 2.36 \times 10^9$ M^{-1}; $\sigma = 13712.62$.

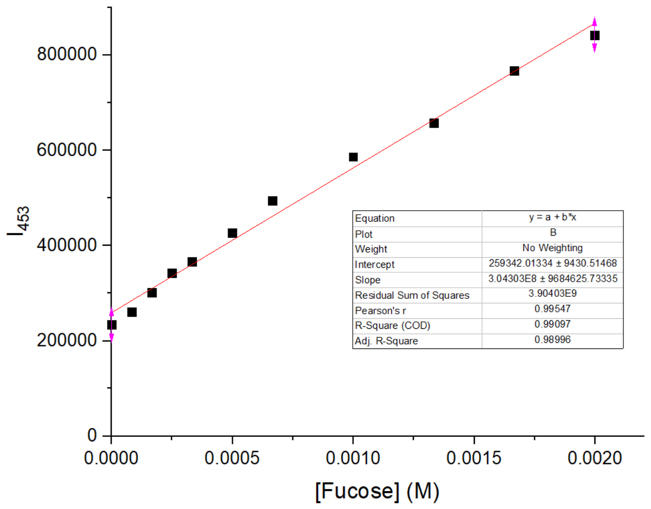

Figure S35. Calibration curve for LOD determination: plot of the fluorescence intensity of receptor **1** versus the fucose concentration in MeOH/10 mM phosphate buffer (80:20); λ_{ex}: 350 nm; λ_{em}: 453 nm; $k = 3.04 \times 10^8$ M^{-1}; $\sigma = 12995.09$.

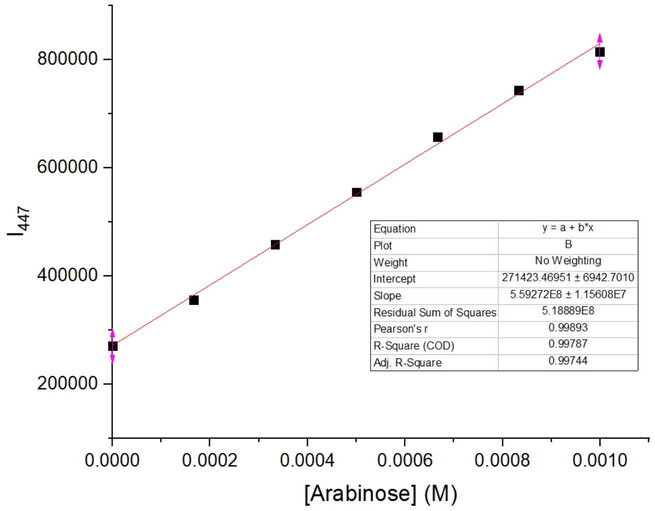

Figure S36. Calibration curve for LOD determination: plot of the fluorescence intensity of receptor **1** versus the arabinose concentration in MeOH/10 mM phosphate buffer (80:20); λ_{ex}: 350 nm; λ_{em}: 447 nm; $k = 5.59 \times 10^{8}$ M^{-1}; $\sigma = 13883.57$.

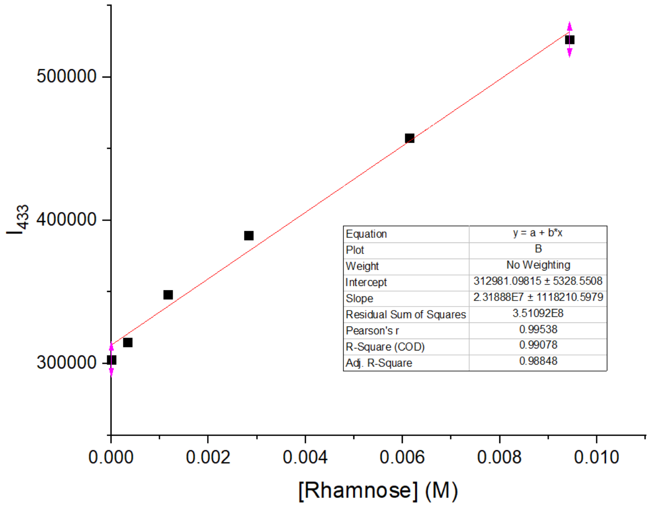

Figure S37. Calibration curve for LOD determination: plot of the fluorescence intensity of receptor **1** versus the rhamnose concentration in MeOH/10 mM phosphate buffer (80:20); λ_{ex}: 350 nm; λ_{em}: 433 nm; $k = 2.31 \times 10^{7}$ M^{-1}; $\sigma = 15759.02$.

9. Fluorescence response of 1 to D-glucose in pure MeOH and MeOH/10mM phosphate buffered-D_2O (80:20)

All fluorescence titration experiments were carried out at 298 K on a Horiba Fluoromax spectrofluorometer. A solution of the receptor (3 mL) at a known concentration (1 μM) in anhydrous methanol (Figure S38) or anhydrous methanol/10 mM phosphate buffered-D_2O (80:20) (Figure S39), was placed in a quartz cuvette (3 mL, 10 mm path length). A solution of D-glucose in phosphate buffered-D_2O solution (pH 7.4, 10 mM) was then added to the solution containing the receptor **1** and the fluorescence spectrum was recorded after each addition. Guest additions to the receptor were performed using a procedure which kept the concentration of receptor constant throughout the titration. The excitation wavelength was fixed at 350 nm for each titration, and the emission spectrum recorded between 400 – 700 nm. All fluorescence titration experiments were carried out three times.

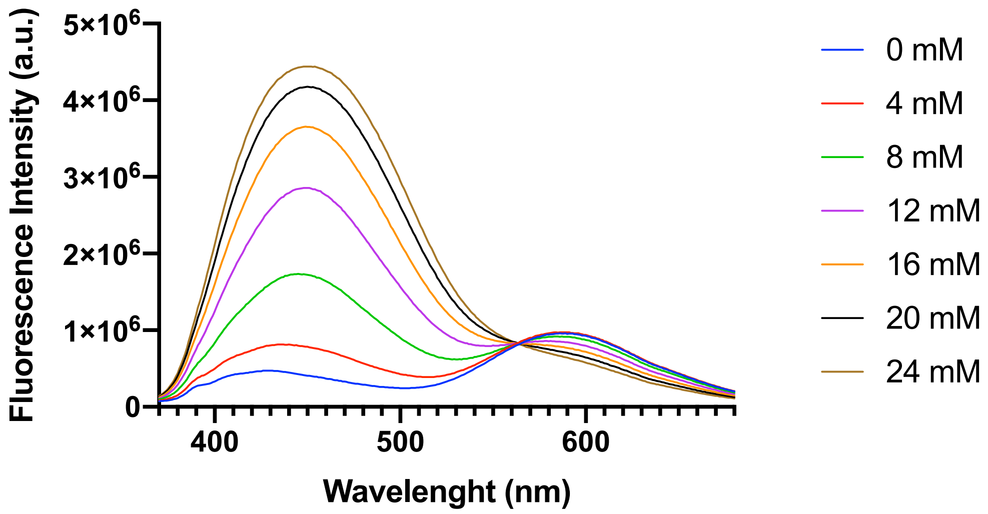

Figure S38. Fluorescence titration of **1** with D-glucose. λ_{ex} = 350 nm; [**1**] = 1 μM; solvent = anhydrous MeOH.

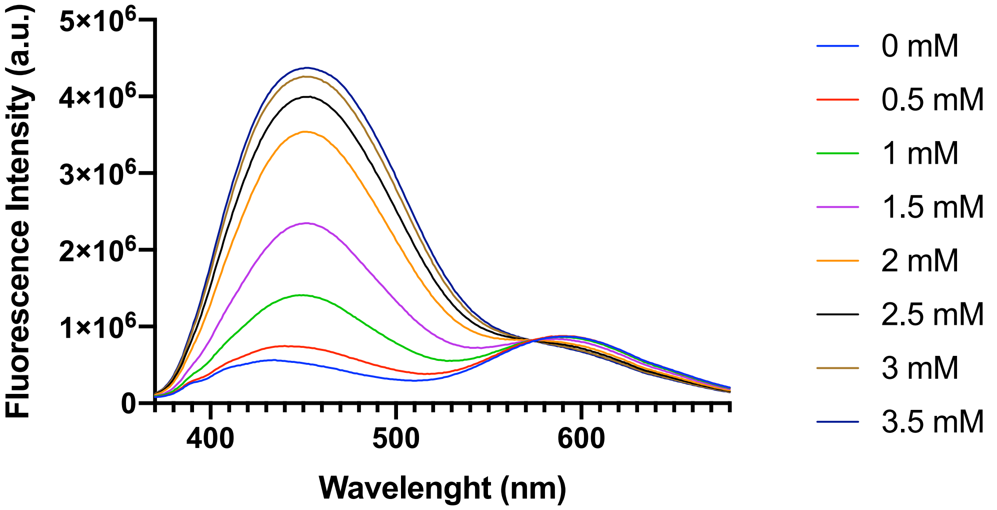

Figure S39. Fluorescence titration of **1** with D-glucose. λ_{ex} = 350 nm; [**1**] = 1 µM; solvent = methanol/10 mM phosphate buffered-D_2O (80:20).

10. References

[1] Chen, W.; Guo, C.; He, Q.; Chi, X.; Lynch, V. M.; Zhang, Z.; Su, J.; Tian, H.; Sessler, J. L. *J. Am. Chem. Soc.* **2019**, *141*, 14798-14806.
